# Sponges-Cyanobacteria associations: Global diversity overview and new data from the Eastern Mediterranean

**DOI:** 10.1371/journal.pone.0195001

**Published:** 2018-03-29

**Authors:** Despoina Konstantinou, Vasilis Gerovasileiou, Eleni Voultsiadou, Spyros Gkelis

**Affiliations:** 1 Department of Botany, School of Biology, Aristotle University of Thessaloniki, Thessaloniki, Greece; 2 Department of Zoology, School of Biology, Aristotle University of Thessaloniki, Thessaloniki, Greece; 3 Institute of Marine Biology, Biotechnology & Aquaculture, Hellenic Centre for Marine Research, Heraklion, Crete, Greece; Museum National d'Histoire Naturelle, FRANCE

## Abstract

Sponge-cyanobacteria associations have attracted research interest from an ecological, evolutionary and biotechnological perspective. Current knowledge is, in its majority, “hidden” in metagenomics research studying the entire microbial communities of sponges, while knowledge on these associations is totally missing for certain geographic areas. In this study, we (a) investigated the occurrence of cyanobacteria in 18 sponge species, several of which are studied for the first time for their cyanobionts, from a previously unexplored eastern Mediterranean ecoregion, the Aegean Sea, (b) isolated sponge-associated cyanobacteria, and characterized them based on a polyphasic (morphological-morphometric and molecular phylogenetic analysis) approach, and (c) conducted a meta-analysis on the global diversity of sponge species hosting cyanobacteria, as well as the diversity of cyanobacterial symbionts. Our research provided new records for nine sponge species, previously unknown for this association, while the isolated cyanobacteria were found to form novel clades within *Synechococcus*, Leptolyngbyaceae, Pseudanabaenaceae, and Schizotrichaceae, whose taxonomic status requires further investigation; this is the first report of a Schizotrichaceae cyanobacterium associated with sponges. The extensive evaluation of the literature along with the new data from the Aegean Sea raised the number of sponge species known for hosting cyanobacteria to 320 and showed that the cyanobacterial diversity reported from sponges is yet underestimated.

## Introduction

Sponges (phylum Porifera) are among the oldest Metazoa and constitute a reservoir of exceptional microbial diversity [1, 2]. Sponge-associated microbes, which include bacteria, archaea and unicellular eukaryotes, played key roles in the evolution and ecological success of sponges [3, 4]. Microbes can comprise up to 40% of the sponge volume, with cyanobacteria often representing the most common and most important inhabitants [4, 5]. The sponge-cyanobacteria association has been characterized as a mutualistic interaction, in which sponge hosts may supply a shelter habitat for cyanobacteria, and cyanobacterial symbionts can provide sponges with supplemental nutrition (by means of photosynthesis and nitrogen fixation), chemical defence (by production of bioactive compounds) and UV protection ([5, 6] and references therein). Cyanobacterial symbionts may be acquired vertically, from parent to offspring (through larvae), horizontally, from the surrounding environment, or by both transmission routes [5].

Cyanobacteria, including both coccoid [6–10] and filamentous [11–13] morphs, have been reported in association with more than 100 sponge species from both tropical and temperate regions [5, 14]. Next-generation sequencing and advances in genomics continuously provide the research field of sponge-microbes associations with new data, and reveal new associations of cyanobacteria with sponges [15–19]. However, a detailed account of sponge-cyanobacteria associations is currently not available, although this would be very useful as a basis for future research purposes.

While molecular metagenomic sequencing can provide some information independent of our ability to culture bacteria, a true understanding of the physiology of these organisms and their roles in host health and ecology, along with natural product biosynthesis requires their cultivation in the laboratory [20]. An important fraction of microbial diversity is harboured in individual strains, therefore identification of conspecific bacterial strains is imperative for improved understanding of microbial community functions [21]. Besides, strains held in culture collections are pivotal for comparative purposes in current taxonomic or phylogenetic studies of prokaryotes in general, and cyanobacteria in particular [22]. Furthermore, comparative genomic studies, based on cyanobacteria strains, have shed light on biosynthetic mechanisms unique to cyanobacteria or rarely described from other organisms and revealed novel natural product diversity [23]. Therefore, strain isolation is important in both understanding the diversity of cyanobionts and exploring their biotechnological potential.

Knowledge of the diversity of sponge-associated cyanobacteria is of great scientific interest in order to understand the ecology and evolutionary history of their symbiotic relationships [6]. In fact, closely related cyanobacterial symbionts may play different key roles in the symbiotic interaction with sponges [6, 9]. All the above underline the importance of exploring these associations and providing baseline information on the diversity of cyanobacterial associates, especially in areas poorly studied from this point of view.

Regarding sponge diversity and ecology, the Aegean Sea constitutes the most thoroughly studied marine area of the Eastern Mediterranean Sea, and one of the best studied in the entire Mediterranean Sea [24, 25, 26]. Τhe species list of cyanobacteria has been recently published for this area [27], but no information exists on the associations of sponges with cyanobacteria.

The present study aims to provide (a) information on the occurrence and diversity of cyanobacteria associated with sponge species in the Aegean Sea, for the first time, using mainly culture-based approach and (b) an up to date overview of the global cyanobacterial diversity reported from sponges through an extensive study of the existing data.

## Materials and methods

### Sampling and sample processing

Sampling was performed in Kassandra, Chalkidiki Peninsula, Greece (39.951° N, 23.685° E) in order to collect typical sponge species and morphotypes of the North Aegean Sea rocky sublittoral zone; this site is well-known for its rich sessile communities [28]. Samples were collected in October 10th, 2014 within an 80-min SCUBA dive, from rocky reefs, vertical walls and overhangs, at a depth range of 6.8–21 m. Water temperature at all sampling depths was 22°C (measured with a SUUNTO dive computer). Rocky reefs were covered by assemblages of the phaeophytes *Padina pavonica* and *Dictyota dichotoma*, and the chlorophyte *Halimeda tuna*, while vertical walls and overhangs were covered with coralligenous assemblages, dominated by the rhodophytes *Mesophyllum* spp. and *Peyssonnelia* spp., sponges, and the scleractinian coral *Madracis pharensis*.

**Fig 1:**
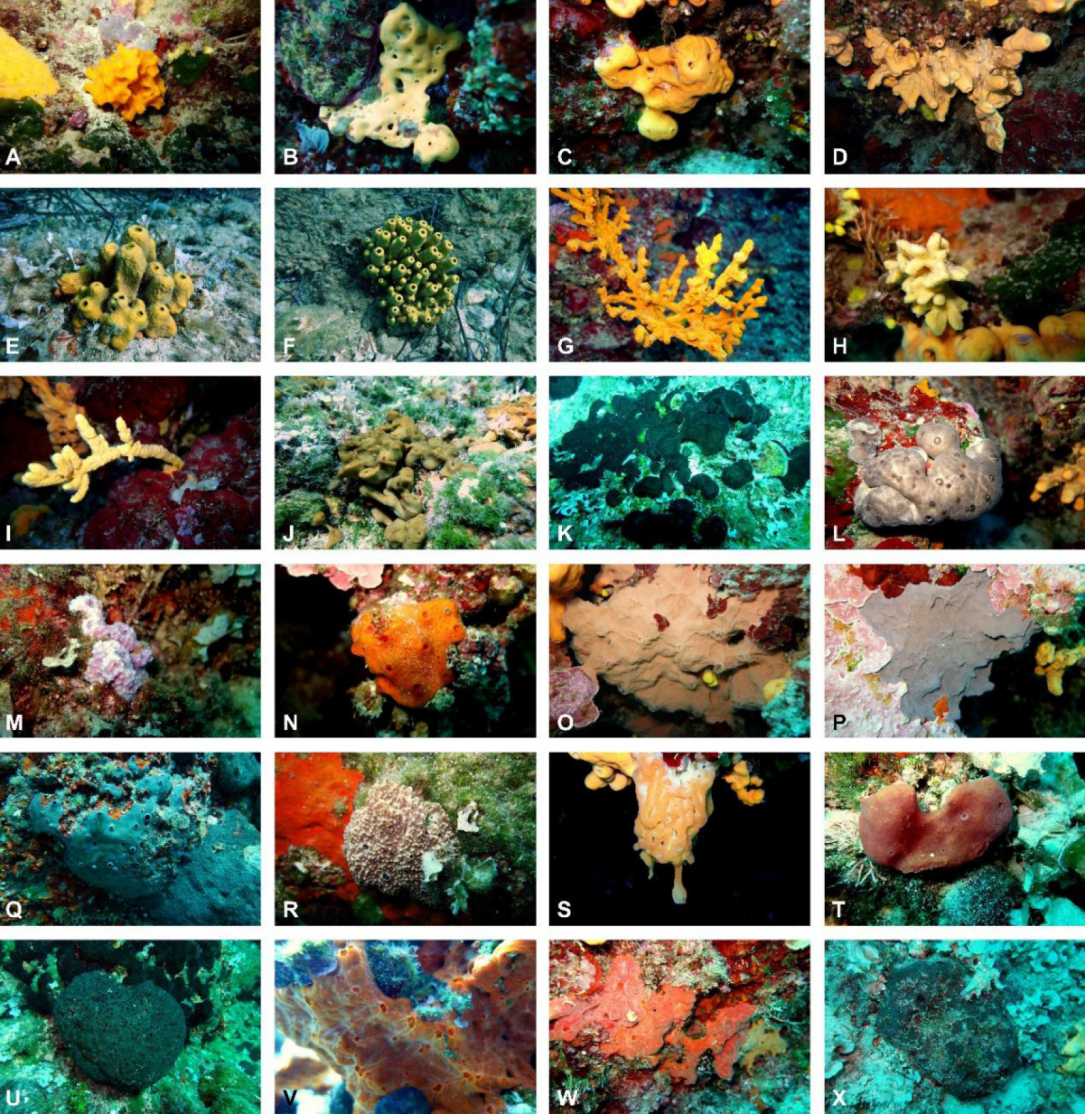
Sponge specimens collected in the framework of this study. (A) *Acanthella acuta*, (B), *Agelas oroides* specimen 1, (C) *A*. *oroides* specimen 2, (D) *A*. *oroides* specimen 3, (E) *Aplysina aerophoba* specimen 1, (F) *A*. *aerophoba* specimen 2, (G) *Axinella cannabina*, (H) *Axinella damicornis*, (I) *Axinella verrucosa*, (J) *Chondrilla nucula*, (K) *Chondrosia reniformis* specimen 1, (L) *C*. *reniformis* specimen 2, (M) *Dysidea avara*, (N) *Haliclona (Halichoclona) fulva*, (O) *Hexadella racovitzai* specimen 1, (P) *H*. *racovitzai* specimen 2, (Q) *Ircinia oros*, (R) *Ircinia variabilis*, (S) *Oscarella* sp., (T) *Petrosia (Petrosia) ficiformis*, (U) *Sarcotragus foetidus*, (V) *Spirastrella cunctatrix* specimen 1, (W) *S*. *cunctatrix* specimen 2, (X) *Stryphnus ponderosus*. Photos by V. Gerovasileiou.

Small pieces (≈2x3 cm) of the most conspicuous sponge species were cut with single-use knives, different for each specimen, and placed in separate 25 ml vials. A total of 24 samples were collected (Fig 1, S1 Table). In certain cases, multiple samples of the same species were taken, representing different replicates, morphotypes (i.e. growth form and colour), and depth zones. The most striking example was that of the species *Agelas oroides*, one of the most conspicuous sponges in sciaphilic assemblages of Mediterranean rocky substrata. Typically, *A*. *oroides* has a massive growth form but in semidark cryptic habitats—at least in the Aegean Sea—it may develop as massive-tubular [29]. In this study, three samples of *A*. *oroides* were collected: a massive specimen from an exposed rocky surface at 9.6 m (Fig 1B), a second massive one from a wall surface at 16.7 m (Fig 1C) and one massive-tubular specimen growing few meters apart (Fig 1D), in a nearby semidark overhang.

Sponge samples were transferred in an insulated cooler to the laboratory within 2h and were processed directly. Specimens were washed three times with sterile seawater and were briefly rinsed in 70% (v/v) ethanol to remove planktonic or loosely associated microorganisms according to standard protocols [2]. Specimens were either processed for isolation and fluorescent microscopy or stored at -20^o^ C for the molecular analyses.

Sponge species identifications were confirmed by microscopic examination of morphological characters based on a large number of publications, in accordance with the classification proposed in Systema Porifera [30] and the World Porifera Database [31].

### Fluorescent microscopy

Sections of sponge samples were cut as thinly as possible using a hand-held razor and examined with fluorescent microscopy using a Zeiss Axio imager z2 microscope. Orange-red light (Alexa Fluor 546 filter) detecting autofluorescence of cyanobacterial pigments (phycoerythrin) indicated the presence of cyanobacteria.

### Isolation, cultivation and morphological identification of strains

An 1 cm^3^ portion of each sponge sample was cut into thin sections and homogenised with a mortar and pestle. Serial dilutions of the suspension were prepared in liquid MN medium [32]. The cultures were incubated at 22 ± 1.0°C at a photosynthetic photon flux density of 20 μmol m^-2^ s^-1^ provided by cool white light fluorescent lamps and a light cycle of 12:12h. Monospecific cultures were isolated and further purified by successive transfers and using antibiotics (such as cycloheximide and ampicillin) as described in Rippka [33]. The isolates were deposited in Aristotle University of Thessaloniki Microalgae and Cyanobacteria Collection (TAU-MAC) [34] and can be accessed at http://cyanobacteria.myspecies.info/.

Morphological examination of cyanobacteria isolates was performed using a Zeiss Axio imager z2 microscope. Strains were identified using the taxonomy books by Komárek and Anagnostidis [35, 36]. Microphotographs were taken with an Axio Cam MRc5 digital camera (Carl Zeiss, Germany). Mean cell or filament dimension was calculated after measuring the dimensions of at least 50 individuals (cells or filaments) of each species.

### DNA extraction, amplification and sequencing

DNA extraction was performed according to Atashpaz et al. [37] for cyanobacteria isolates and sponge samples. The amplification of 16S rDNA of cyanobacteria strains and sponge-cyanobacteria associates was done using three sets of cyanobacterial-specific primers: CYA106F / 23S30R, 16S27F /23S30R, CYA106F /CYA781R(a), (b) [38, 39] (S2 Table). The polymerase chain reaction (PCR) of 16S rRNA gene was carried out using an Eppendorf MasterCycler Pro (Eppendorf). All PCR reactions were prepared in a volume of 25 μL containing 5X PCR buffer, 200 μM MgCl_2_, 0.2 mM of each deoxynucleotide triphosphate, 0.5 μΜ of each of the primers, 0.8 U of taq DNA polymerase, and 30–80 ng of genomic DNA, determined with the Nanodrop 2000 (Thermo Scientific, USA). For CYA106F/781R(a),(b) pair PCR conditions were: denaturation for 5 min at 94°C, followed by 36 cycles of denaturation at 94°C for 30 s, annealing at 64.8°C for 30 s and extension at 72°C for 30 s and by final elongation for 5 min at 72°C. For CYA106F/23S30R and 16S27F/23S30R pairs PCR conditions were: denaturation for 4 min at 94°C, followed by 36 cycles of denaturation at 94°C for 30 s, annealing at 57°C for 45 s and extension at 72°C for 2 min and by final elongation for 4 min at 72°C. In order to amplify 16S rRNA from sponges samples, multiple PCR amplification efforts and optimization strategies (modifying buffer, primer, and Mg^2+^ concentration, annealing temperature and PCR cycle number) were performed; furthermore, several dilutions of the extracted DNA were used for the removal of PCR inhibitors. PCR products were separated by 1.5% (w/v) agarose gel in 1X TAE buffer. The gels were stained with Μidory Green (Nippon Genetics Europe GmbH) and photographed under UV transillumination.

PCR products of cyanobacteria strains were purified using the Nucleospin Gel and PCR-clean up (Macherey-Nagel, Germany) kit. The purified products were sequenced using the primers CYA106F/16S27F/23S30R. Sequence data were obtained by capillary electrophoresis (CeMIA SA., Greece) using the BigDye Terminator v3.1 Cycle Sequencing kit (Applied Biosystems Inc., USA). Partial 16S rRNA sequence data were obtained from nine sponge-associated cyanobacteria strains and processed with the BioEdit (Ibis Biosciences 1997–2015) software. Chimera check was performed for sequences using Pintail [40]. The sequences were deposited in Gen Bank database under the accession numbers KY744806-KY744814.

### Phylogenetic analysis

All our new sequences were blasted and the closest relative(s) for each sequence were included in the phylogenetic trees. For the phylogenetic analyses, we selected sequences (>900 bp) belonging to non-heterocytous taxa to examine phylogenetic position of our strains. Multiple sequence alignments were conducted using the CLUSTAL W program [41]. Phylogenetic trees were constructed in Mega 6.06 [42] using (1) the Neighbour-Joining (NJ) method on a Jukes and Cantor distance matrix model, (2) the Maximum-Likelihood (ML) applying a GTR + G + I model of nucleotide substitution. The robustness of the inferred phylogenies was determined by bootstrap analysis based on 1000 resamplings of data.

Phylogenetic relationships among coccoid and filamentous cyanobacteria strains were examined separately. For coccoid cyanobacteria we included free-living *Synechococcus* strains and the closely related *Prochlorococcus*, clones of the specific sponge symbiont “*Candidatus Synechococcus spongiarum”* and representatives of all the genera of Pleurocapsales order. For filamentous cyanobacteria we included marine filamentous cyanobacteria strains of Synechococcales order and almost all available sequences of the genera *Symploca* and *Schizothrix*. To ascertain the precise phylogenetic position of our filamentous strains, we conducted a detailed phylogenetic analysis employing a comprehensive selection of 16S rRNA gene sequences from all genera of Leptolyngbyaceae family, the closely affiliated Heteroleibleiniaceae (the only available sequence belongs to *Tapinothrix*) and sequences from Pseudanabaenaceae.

### Literature data collection

**Fig 2:**
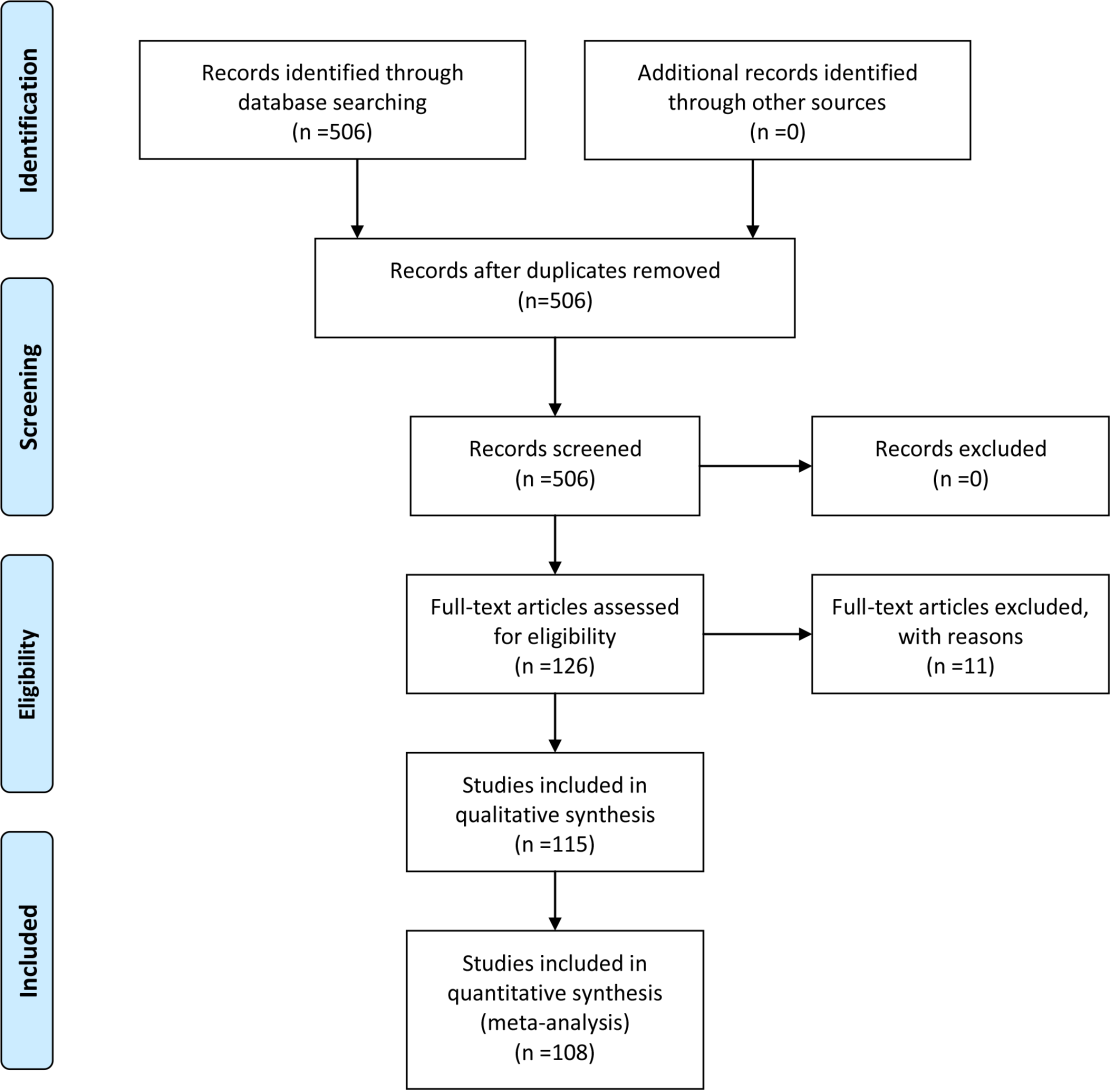
PRISMA 2009 flow diagram. Flow diagram illustrating the search strategy for obtaining the available data on the diversity of cyanobacteria associated with sponges.

In order to obtain the available information on the diversity of cyanobacteria associated with sponges, all relevant literature was searched in Scopus and Web of Science Databases using the keywords: sponge AND cyanobacteria. All published data on sponges harbouring cyanobacteria (given both in the main texts and supplementary files of scientific papers, as well as in online sequence databases) were incorporated into a Microsoft Excel database, including the associated cyanobacteria taxa, along with the study area and methods used for their detection. Data on cyanobacteria colonizing dead and/or infected sponges was not considered since they might be opportunistic organisms that had settled after tissue necrosis (see di Camillo et al. [43]). The taxonomic status of the recorded sponge and cyanobacteria taxa was checked and updated, where necessary, using the World Register of Marine Species [44] and the AlgaeBase [45], respectively. The cyanobacterial symbiont “*Aphanocapsa feldmannii*” was updated and assigned to *Synechococcus* genus according to Usher et al. [46]. The PRISMA flowchart describing the included/excluded literature is given in Fig 2, and the PRISMA checklist in S1 Text [47].

In order to evaluate the scientific research effort devoted to sponge-cyanobacteria association worldwide, scientific studies were classified according to the year of publication and region, while regional data were classified in provinces (worldwide) and ecoregions (only for the Mediterranean Sea) according to Spalding et al. [48]. The number of sponge species of each sponge order was extracted from the online World Porifera Database [31].

## Results and discussion

### Cyanobacteria associated with Aegean sponges

The 24 collected sponge specimens were assigned to 17 Demospongiae and 1 Homoscleromorpha species (Fig 1, S1 Table). The identified sponge species are among the most common Aegean Porifera [49] and form characteristic elements of the Mediterranean benthic communities from which they were collected [50]. Fluorescence microscopy revealed the presence of sponge-associated cyanobacteria in 21 sponge specimens (16 sponge species) (S1 Table). These cyanobacteria were mostly coccoid; filamentous morphs were observed only in three sponge species. The 16S rRNA gene of sponge-associated cyanobacteria was amplified in 12 of the 24 samples; lack of amplification was attributed to biases or errors of PCR, such as inhibition of the PCR by substances co-extracted with the nucleic acids [51], primer mismatch and annealing temperature [52].

Nine sponge species (*Acanthella acuta*, *Axinella cannabina*, *A*. *damicornis*, *A*. *verrucosa*, *Haliclona fulva*, *Hexadella racovitzai*, *Oscarella* sp., *Spirastrella cunctatrix*, and *Stryphnus ponderosus*) were found for the first time as host for cyanobacteria. These species have an Atlanto-Mediterranean distribution, except for *A*. *cannabina*, which is a Mediterranean endemic, more common in the eastern basin [31], an area poorly investigated for sponge-cyanobacteria associations. Cyanobacteria have been previously found living in association with other sponges species studied here such as *Agelas oroides*, *Aplysina aerophoba*, *Chondrilla nucula*, *Dysidea avara*, *Petrosia ficiformis* and *Ircinia variabilis* (e.g., [13, 53, 54]).

**Fig 3:**
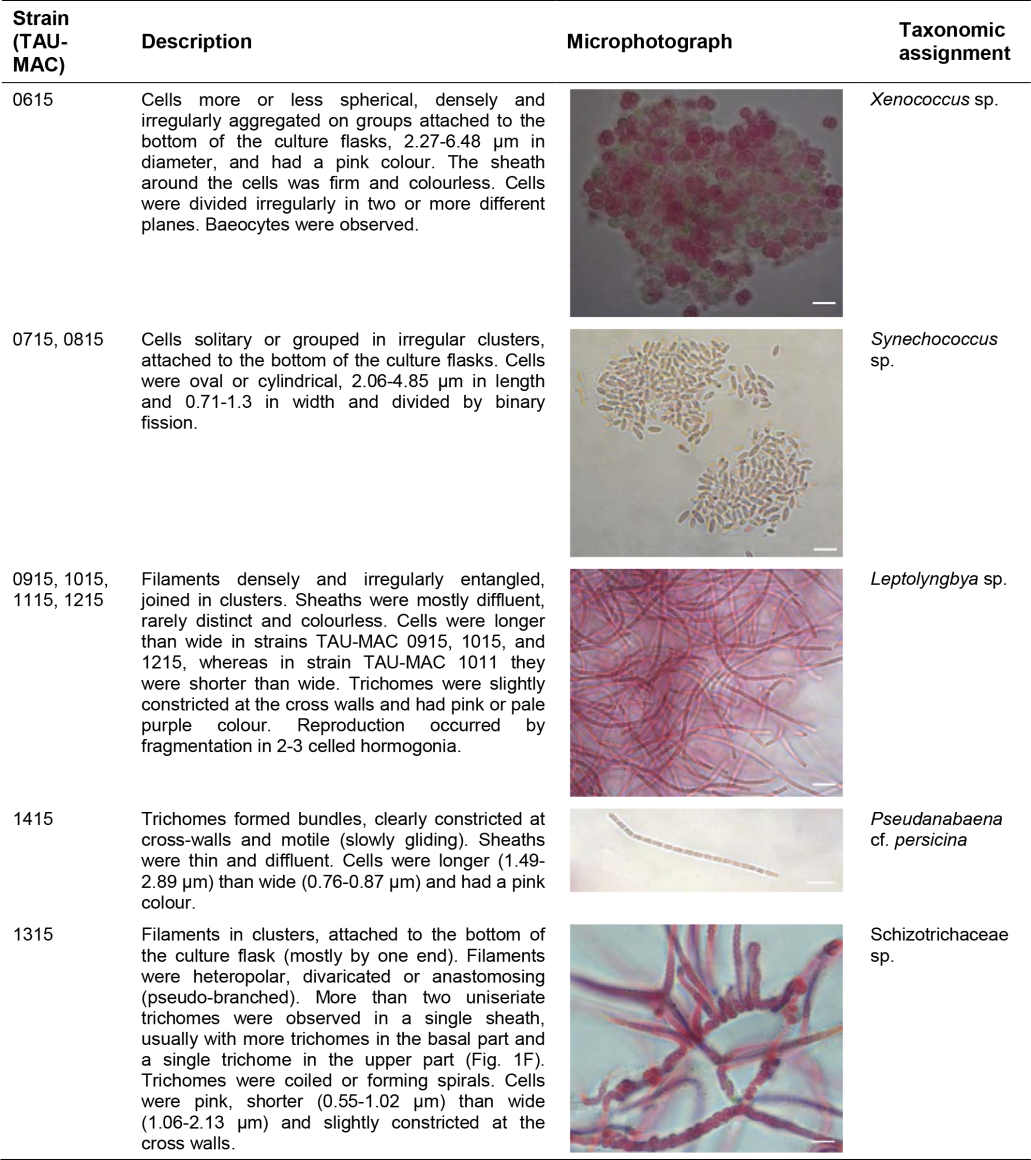
Morphometric and morphological characteristics of the sponge-associated strains isolated from the Aegean Sea. Scale bar, 10 μm. See S1 Appendix for detailed description of the strains.

**Fig 4:**
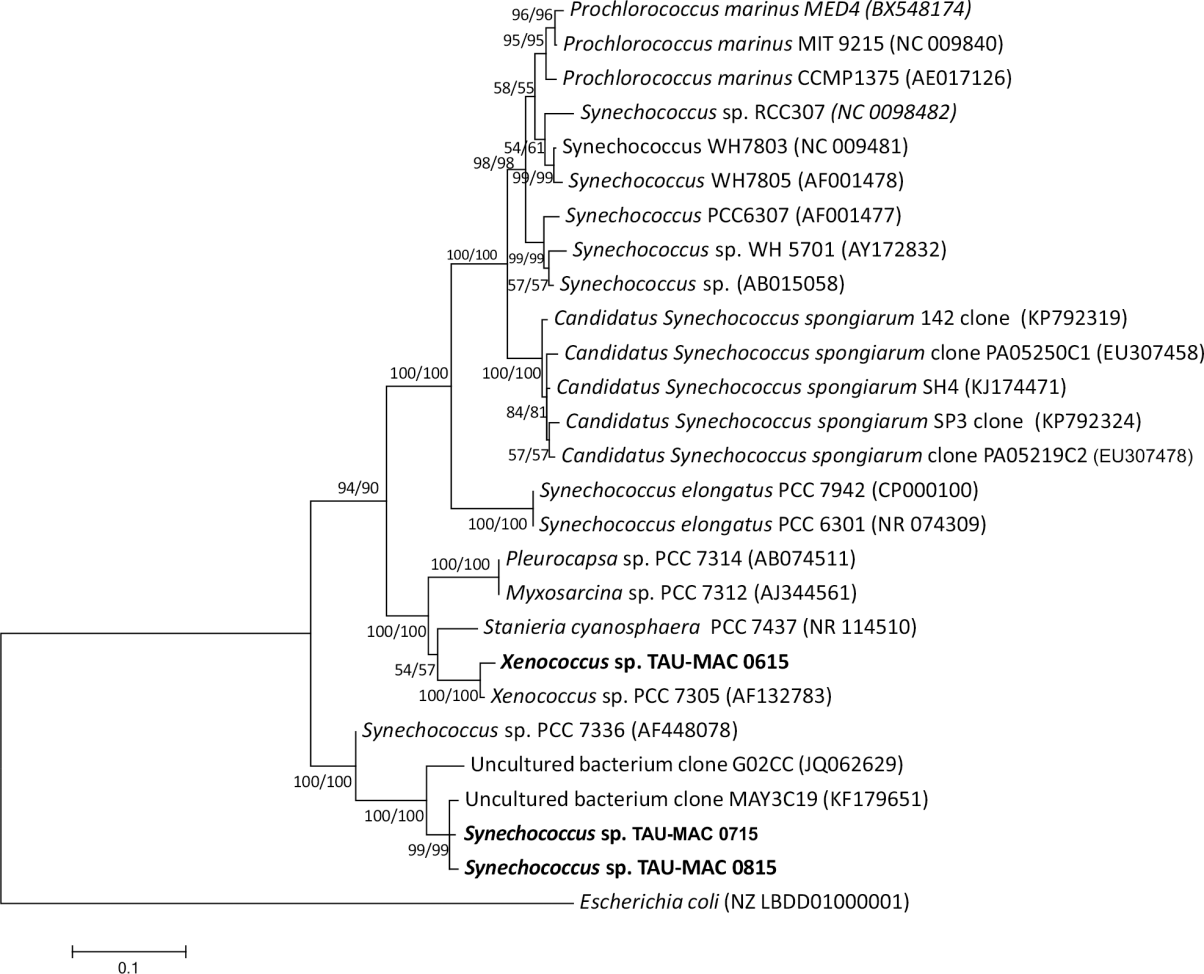
Phylogenetic tree based on 16S rRNA gene sequences of coccoid cyanobacteria and reconstructed using the Maximum-Likelihood (ML) analysis. Numbers above branches indicate the bootstrap value (as percentages of 1,000 replications) for NJ/ML methods. The topologies of ML and NJ analyses were the same. Strains of the present study are indicated in bold, GenBank accession numbers are indicated in brackets. Bar represents 0.1 nucleotide substitutions per site.

**Fig 5:**
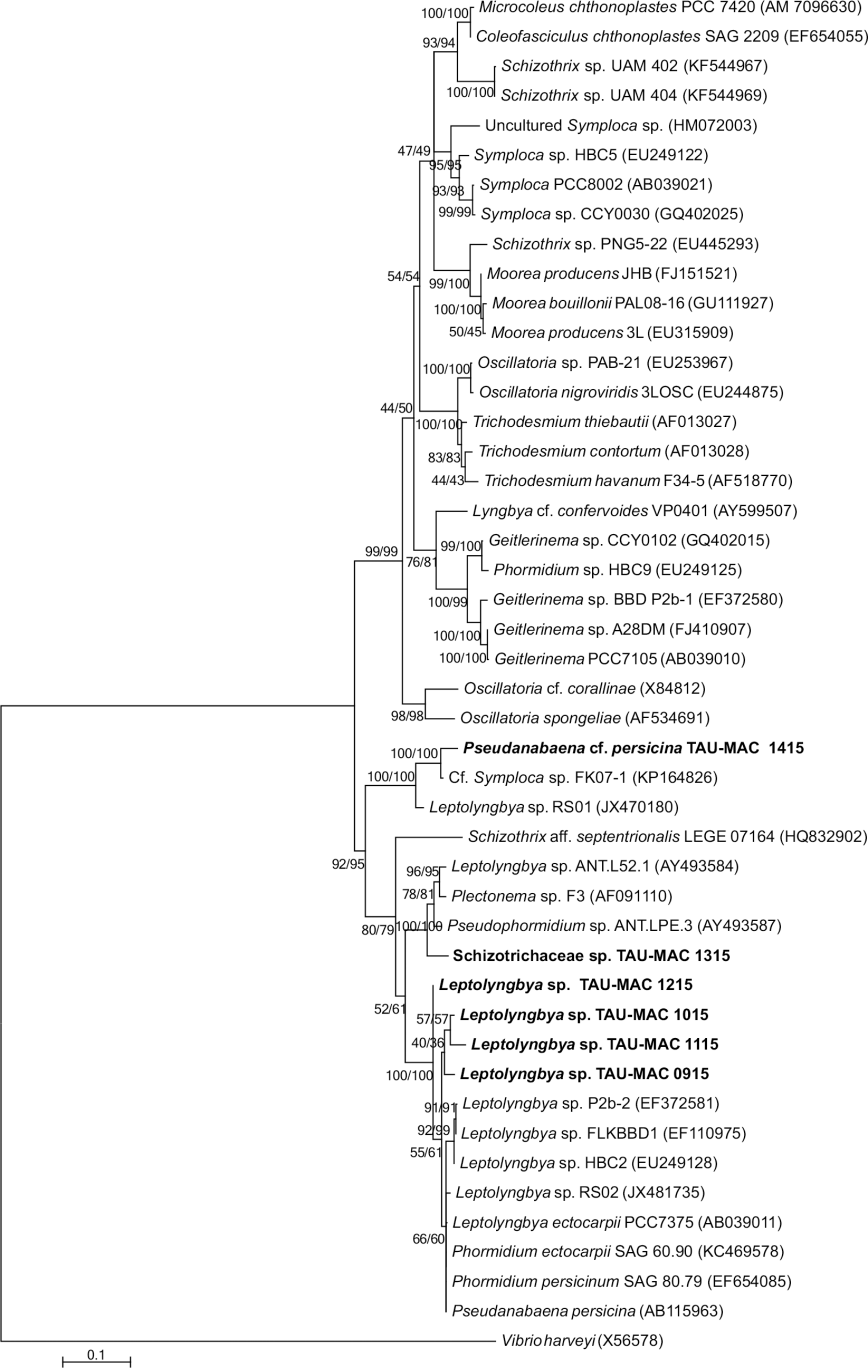
Phylogenetic tree based on 16S rRNA gene sequences of filamentous cyanobacteria reconstructed using the Maximum-Likelihood (ML) method. Numbers above branches indicate the bootstrap value (as percentages of 1,000 replications) for NJ/ML methods. The topologies of ML and NJ analyses were the same. Strains of the present study are indicated in bold, GenBank accession numbers are indicated in brackets. Bar represents 0.1 nucleotide substitutions per site.

We isolated nine cyanobacterial strains, six filamentous and three coccoid, from eight sponge species (S3 Table). Based on morphological traits (Fig 3) and phylogenetic analysis of the 16S rRNA gene (Figs 4 and 5) we classified the strains to *Xenococcus* sp., *Synechococcus* sp., *Leptolyngbya* sp., *Pseudanabaena* cf. *persicina*, and *Schizotrichaceae* sp. (detailed description of the strains is given in S1 Appendix). According to the literature review, only 19 cyanobacteria strains have been isolated to date from the sponge species *Petrosia ficiformis*, *Rhopaloeides odorabile*, *Lamellodysidea herbacea*, *Candidaspongia flabellata*, *Terpios hoshinota*, and *Aplysina cauliformis* [11, 55, 56, 57]; these strains were assigned to the genera *Leptolyngbya*, *Synechococcus*, *Cyanobium*, *Halomicronema*, and *Oscillatoria*, whereas nine of them remain unidentified [58]. Therefore, a total of 28 cyanobacteria strains isolated from 12 sponge species are presently known (S3 Table).

*Xenococcus-*like cyanobacteria have been previously found in association with the demosponge *Hymeniacidon perlevis* [59]. *Ircinia variabilis* is known for its association with coccoid cyanobacteria *Synechococcus*/*Synechocystis* [13, 46, 60, 61, 62]. *Synechococcus* sp. (strains TAU-MAC 0715 and 0815) were isolated from the sponges *A*. *cannabina* and *A*. *damicornis*, respectively. This is the first report of cyanobacteria associated with these sponge species.

*Leptolyngbya* sp. (strains TAU-MAC 0915, 1015, 1115, and 1215) were isolated from the sponges *P*. *ficiformis*, *D*. *avara*, *A*. *acuta* and *C*. *nucula*, respectively. Coccoid and filamentous cyanobacteria living in association with *P*. *ficiformis*, *D*. *avara* and *C*. *nucula* have been previously reported [46, 54, 63, 64, 65, 66], whereas no information exists for *A*. *acuta*. *Pseudanabaena* cf. *persicina* (strain TAU-MAC 1415) was isolated from *A*. *damicornis*. The only reports of *Pseudanabaena* living in association with sponges lies in the taxonomic book of Komárek and Anagnostidis [36] for *P*. *persicina* without any further detail, and in Isaacs et al. [67] showing a clone assigned to *Pseudanabaena* derived from *Clathria prolifera*. Our strain showed 87% pairwise sequence similarity with the *Pseudanabaena* clone derived from *Clathria prolifera* [67]; however, due to the small sequence size (<1000 bases) the latter was excluded from our phylogenetic analysis. *Schizotrichaceae* sp. (strain TAU-MAC 1315) was isolated from *A*. *aerophoba*, a well-studied sponge concerning symbiotic interactions with microbes [7, 53, 68, 69]. This is the first report of a filamentous cyanobacterium in association with *A*. *aerophoba*, as only *Synechococcus* is known to live in association with this sponge (e.g., [7, 68, 70]). Moreover, this is the first report of a Schizotrichaceae-like cyanobacterium associated with sponges. Overall, our polyphasic assessment showed that some of the strains form novel clades within *Xenococcus*, *Synechococcus*, *Leptolyngbya*, *Pseudanabaena*, and Schizotrichaceae.

### Overview of sponge-associated cyanobacterial diversity

#### Research effort and advances

**Fig 6:**
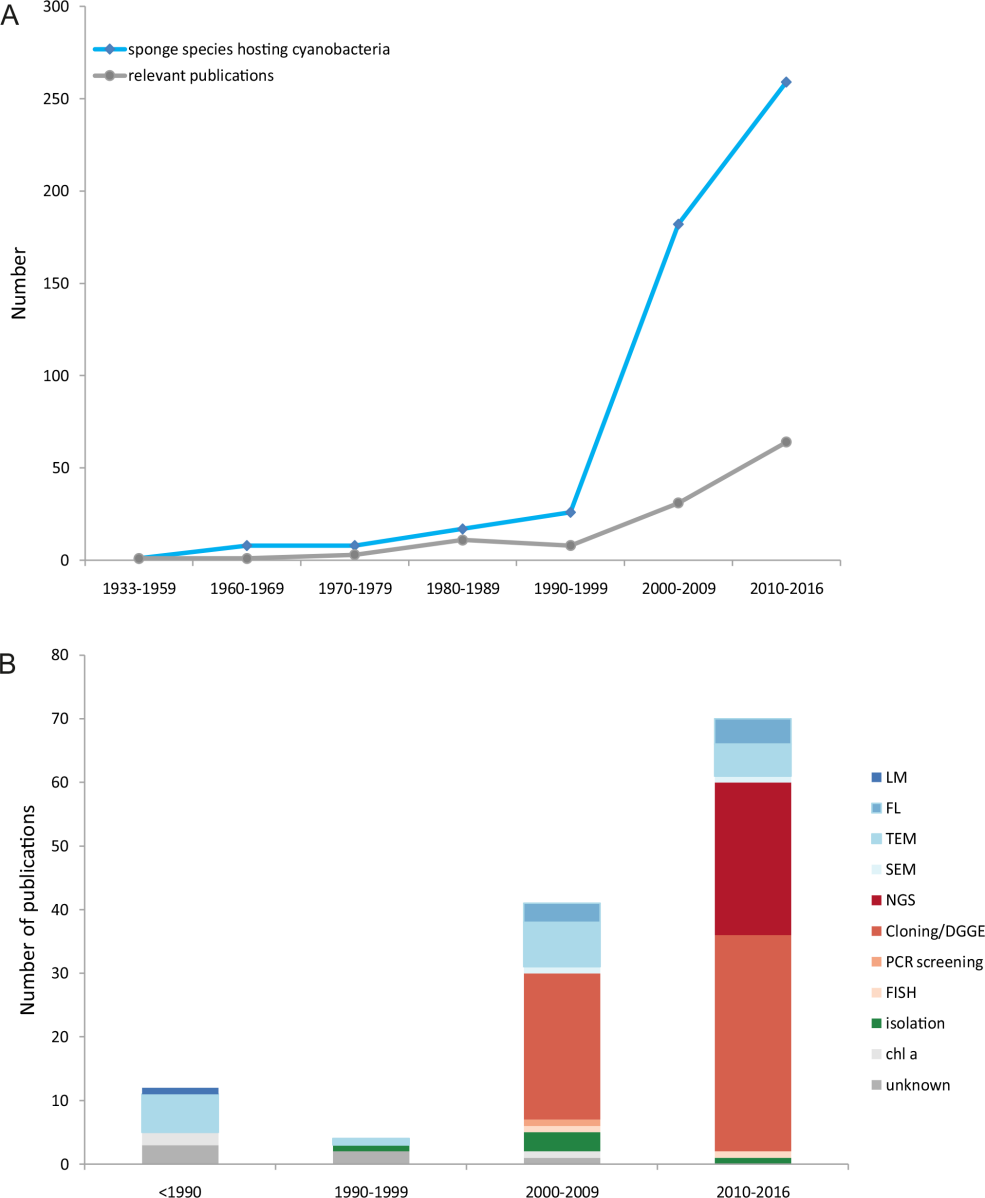
Sponge species hosting cyanobacteria recorded through time (A), and methods used for the investigation of cyanobacteria symbionts (B), in relation with research effort (i.e. number of publications). LM: Light microscopy, FL: Fluorescence microscopy, TEM: Transmission electron microscopy, SEM: Scanning electron microscopy, NGS: Next generation sequencing, DGGE: Denaturing gradient gel electrophoresis, FISH: Fluorescence *in situ* hybridization, chl a: chlorophyll *a* measurements.

The extensive study of the literature yielded 115 publications (Fig 2) on sponge-cyanobacteria associations, covering a time span of 83 years (Fig 6A). The study of this material, alongside with the new data from our research in the Aegean Sea, revealed that, to date, 320 sponge species (S4 Table) have been reported to host cyanobacteria increasing this number by 300% compared to the last review by Diaz et al. [14]. The first record of a cyanobacterium associated with sponges was in 1933 and the second one was published approximately 30 years later [14]. An upward trend was observed over the years for both the number of publications (84.2% appeared after 2000) and the number of sponge species in which cyanobacteria were found (Fig 6A). This trend could be a result of the recent development and use of molecular techniques (cloning, DGGE, NGS) (Fig 6B). Until the late 1990s, the presence of symbiotic cyanobacteria was being detected with the use of microscopy and chlorophyll *a* measurements (e.g., [71, 72]). Molecular tools have not replaced microscopic methods, since both are used nowadays for the study of sponge-associated cyanobacteria (e.g., [59, 73]). Culture dependent methods have been used in few studies (Fig 6B) over the years; it has been estimated that only 0.1–1.0% of the total bacterial cells from host-associated bacterial communities had been isolated [54, 74]. Hardoim et al. [62] showed that cultivation approaches failed to capture the prevalent bacterial associates of marine sponges, but were successful in the cultivation of the least abundant associates which remain elusive to cultivation-independent methods, indicating the importance of using multiple methods for a more comprehensive analysis of associate abundance in marine sponges.

Sponge-cyanobacteria associations have been surveyed mainly for their diversity and ecology, and less attention has been given in evolution, functional aspects and the production of secondary metabolites. Almost 40% of the studies focus exclusively on cyanobacteria, whereas 80% of the research dealing exclusively with sponge-cyanobacteria associations was published before 2010. A great part of the cyanobacteria-sponges associations knowledge is “hidden” in publications studying the entire microbial communities of sponges (e.g., [2, 15]) or individual publications of local level, sometimes available only in the form of DNA sequences in relevant databases (e.g., [16]). Rapid advances in molecular techniques and bioinformatics took place in the last decade (Fig 6B) and made the investigation of evolutionary relationship and functional aspects of this association feasible. Genome-level research on sponge symbionts and their symbiotic mechanisms is still limited, but has already shed light on sponge symbionts and their symbiotic mechanisms: eight genomes of sponge-associated cyanobacteria are available and represent four different clades of “*Ca*. *Synechococcus spongiarum*” which are associated with four different host sponges from three geographic locations [17, 18, 19]. Comparisons of half of those genomes to those of free-living cyanobacteria have revealed general adaptations to life inside sponges and specific adaptations of each phylotype [17, 18].

#### Biogeography

**Fig 7:**
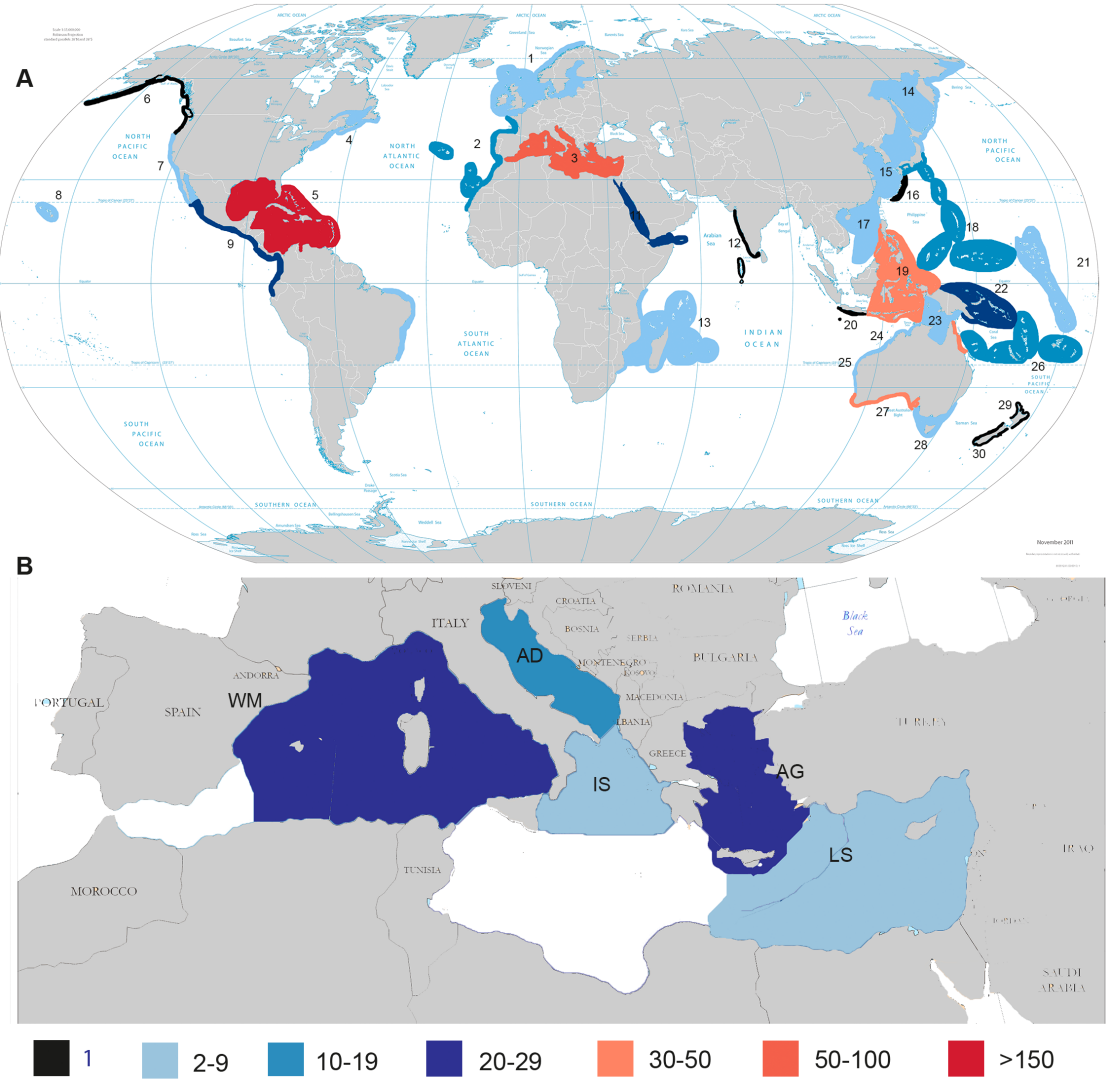
Regional pattern of records of sponge-cyanobacteria associations. Provinces in A and ecoregions in B are according to Spalding et al. [48]: 1, Northern European Seas; 2, Lusitanian; 3, Mediterranean Sea; 4, Cold Temperate Northwest Atlantic; 5, Tropical Northwestern Atlantic; 6, Cold Temperate Northeast Pacific; 7, Warm Temperate Northeast Pacific; 8, Hawaii; 9, Tropical East Pacific; 10, Tropical Southwestern Atlantic; 11, Red Sea and Gulf of Aden; 12, Central Indian Ocean Islands; 13, Western Indian Ocean; 14, Cold Temperate Northwest Pacific; 15, Warm Temperate Northwest Pacific; 16, South Kuroshio; 17, South China Sea; 18, Tropical Northwestern Pacific; 19, Western Coral Triangle; 20, Java Transitional; 21, Marshall, Gilbert, and Ellis Islands; 22, Eastern Coral Triangle; 23, Sahul Shelf; 24, Northwest Australian Shelf; 25, West Central Australian Shelf; 26, Tropical Southwestern Pacific; 27, Southwest Australian Shelf; 28, Southeast Australian Shelf; 29, Southern New Zealand; 30. Northern New Zealand; WM, Western Mediterranean; AD, Adriatic Sea; IS, Ionian Sea; AG, Aegean Sea; LS, Levantine Sea.

The number of records of sponge-cyanobacteria associations differed among geographic areas (Fig 7A). Some marine provinces, such as the Tropical Northwestern Atlantic and Mediterranean Sea, which are among the most species-rich and best studied areas for their sponge fauna in general [75], have been fairly well studied for their sponge-associated cyanobacterial diversity (166 and 55 records of sponge-cyanobacteria associations derived from 27 and 26 publications accordingly), while others have received little attention (Fig 7A). Among Mediterranean ecoregions, the western basin was the best-studied area according to both the records of sponge-cyanobacteria associations (Fig 7B) and the number of publications. The Aegean Sea, previously completely unexplored, hosts 23 records of sponge-cyanobacteria associations, according to the records added by this study (Fig 7B). Usher [6], evaluating ecological aspects of the sponge-cyanobacteria associations, found that the numbers of cyanobacterial symbionts vary both within different areas and within sponge species; she suggested that the nature of the symbiosis is affected by the host species, light levels, predation, sediment load, and other stressors. Erwin and Thacker [7], using ITS phylogeny, revealed 12 distinct clades of “*Ca*. *Synechococcus spongiarum*” structured by both geography and host phylogeny. Erwin et al. [60] showed that Mediterranean *Ircinia* spp. host a specific, novel symbiont clade within “*Ca*. *Synechococcus spongiarum”*. These symbionts exhibited distinct habitat preferences correlated with irradiance and was suggested that this may be an adaptive attribute that allows for flexibility in host–symbiont interactions across the seasonal fluctuations in light and temperature, characteristic of temperate environments [60]. Overall, cyanobacterial associations with sponges appear to be as common in temperate areas as in tropical ones.

#### Cyanobacteria and sponge taxa

**Fig 8:**
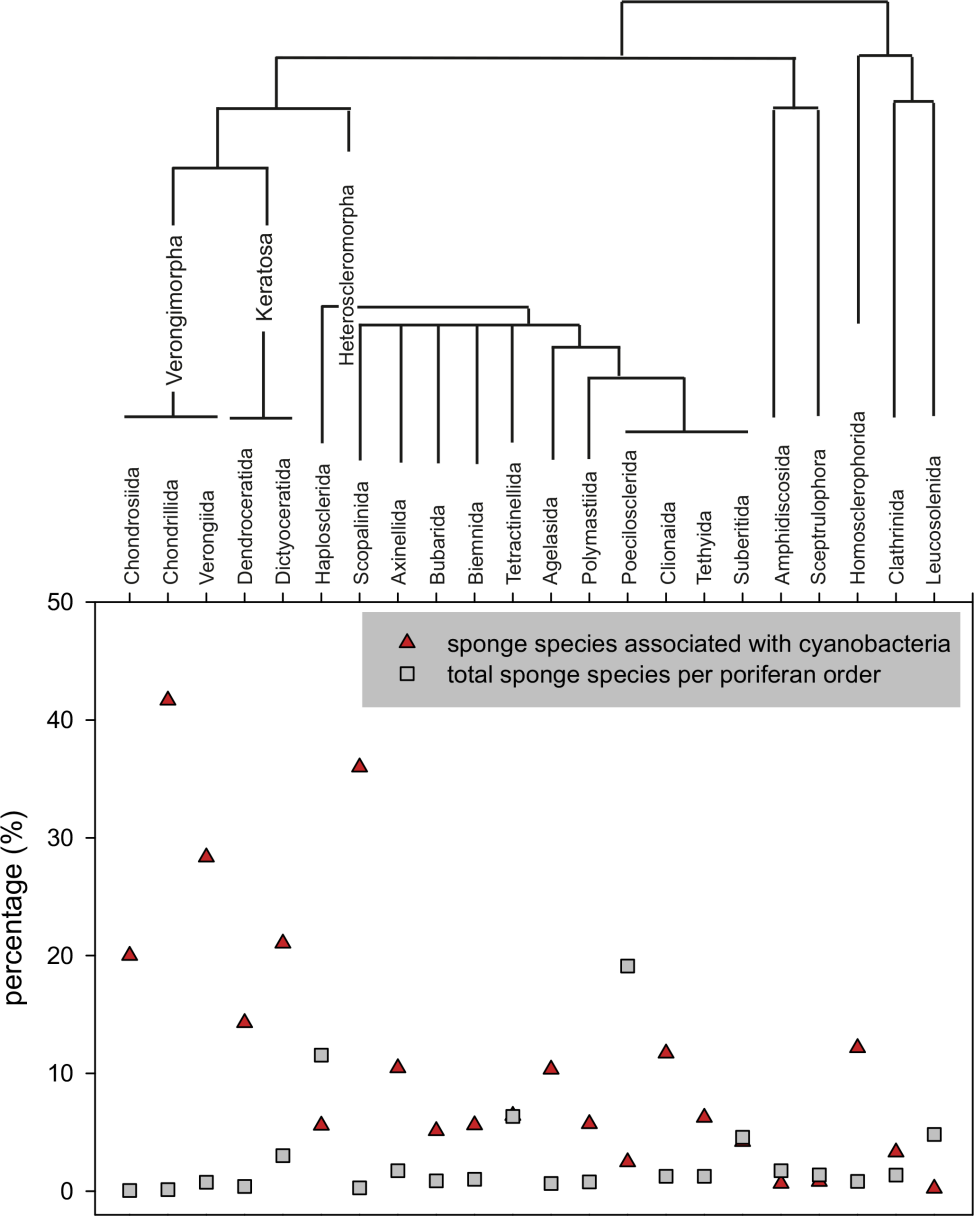
Percentage of sponges hosting cyanobacteria per order and percentage of sponge species per order. Species associated with cyanobacteria: number of sponge species hosting cyanobacteria per order/number of sponge species per order (%); sponge species: number of sponge species per order/total number of known sponge species (%). Cladogram was constructed according to the phylogenetic relationships of sponge orders given in Voigt et al. [79], Morrow and Cardenas [76] and Wörheide et al. [80].

**Fig 9:**
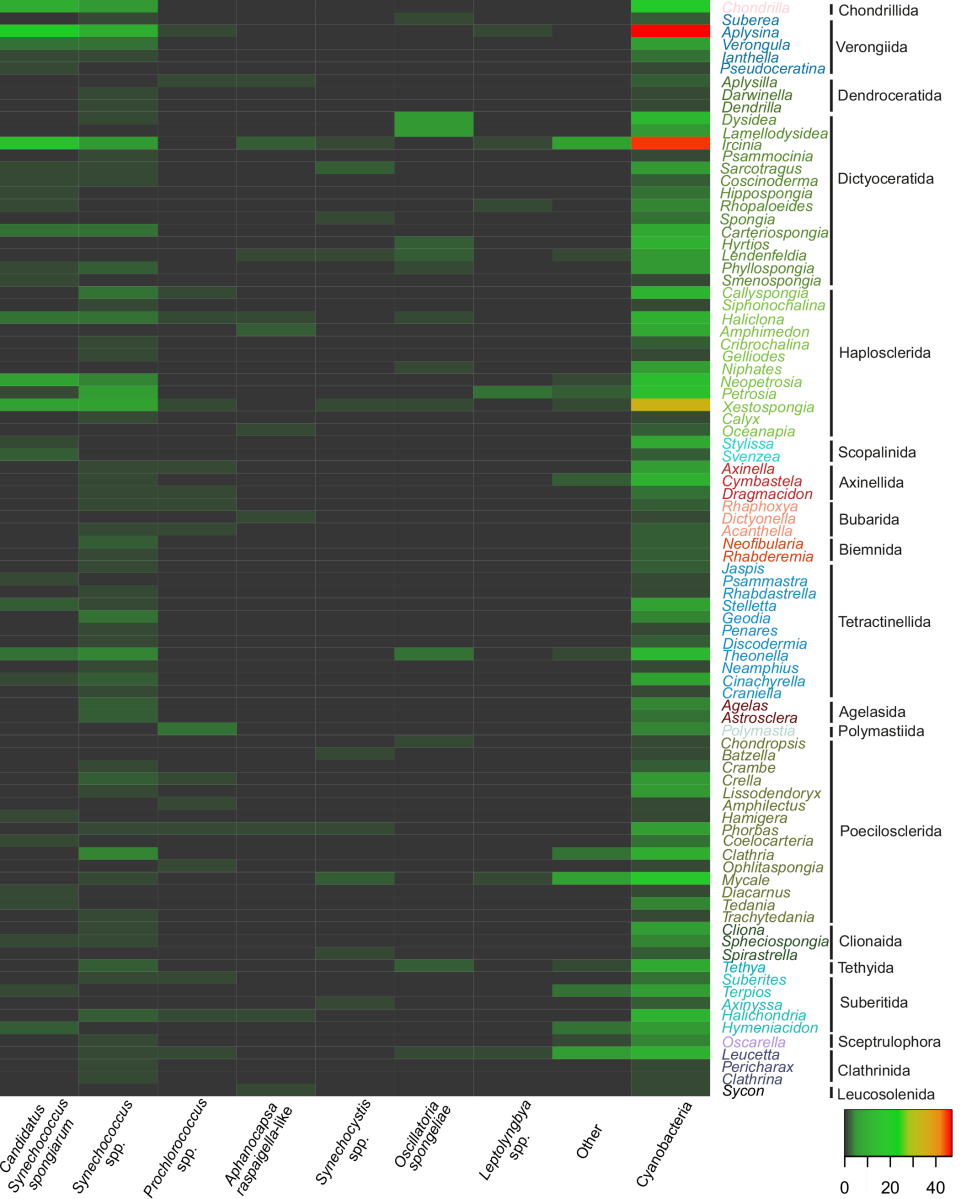
Taxonomic profile of sponge-cyanobacteria associations. Sponge genera are grouped in orders. Sponge orders are presented according to their phylogenetic relationships given in Voigt et al. [79], Morrow and Cardenas [76], and Wörheide et al. [80]. Colour scale shows relative abundance of cyanobacteria records within hosts. Sponge genera where no specific cyanobacterial taxon is reported are shown in S5 Table.

The sponge species reported to live in association with cyanobacteria belonged to 22 orders (Fig 8) and 60 families of the phylum Porifera as follows: 53 families of Demospongiae, 3 families of Calcarea, 2 families of Hexactinellida, and 2 families of Homoscleromorpha (S4 Table). Our review showed that all poriferan classes can host cyanobacteria, while only Demospongiae and Calcarea were known to live in association with cyanobacteria before [5, 14]. Some demosponge orders, such as Chondrosiida, Chondrillida, Verongiida, Dictyoceratida and Dendroceratida appear to harbour cyanobacteria more frequently than others (Fig 8), although they are among the less numerous in species number, with less than 3% of the total demosponges each. As seen in Fig 8, these orders belong to the phylogenetically close subclasses Verongimorpha and Keratosa [76]. On the other hand, our results showed that Haplosclerida and Poecilosclerida, with large numbers of sponge species (>10% representation in the total sponge species number), appear to rarely form associations with cyanobacteria. The higher number of recorded associations among cyanobacteria and certain demosponge orders could be due to preferences for certain sponge taxa such as *Ircinia* and *Aplysina* (Fig 9). The fact that these sponge taxa and more generally Dictyoceratida and Verongiida live preferably in warm and temperate areas [77, 78] indicates a possible bioclimatic pattern deserving further investigation.

Concerning cyanobacterial diversity in sponge species, the literature review showed that in almost half of the cases, the occurrence of cyanobacteria was generally reported (Fig 9), without any further taxonomic identification. This could be attributed to the fact that only phylum-wide investigations were carried out in recent studies (e.g., [2, 15]). Coccoid cyanobacteria morphs (*Synechococcus* spp., *Prochlorococcus* spp., *Aphanocapsa* sp. and *Synechocystis* spp.) were found in sponges more frequently than the filamentous morphs (*Oscillatoria spongeliae* and *Leptolyngbya* spp.) (Fig 9, S5 Table). *Synechococcus* proved to be a widely distributed genus, extensively investigated in marine environments where, along with *Prochlorococcus* [81], they represent the most abundant autotrophs (e.g., [82]). The widespread symbiont “*Ca*. *Synechococcus spongiarum*” was highly represented (18%) among cyanobacterial associates. Up to date, there is no evidence that particular sponge species host particular cyanobacterial symbionts, despite geographic separation [6]. The major clades of cyanobacterial symbionts, “*Ca*. *Synechococcus spongiarum”*, *Synechococcus* spp., and *O*. *spongeliae*, are not specific to any particular sponge species (Fig 9) and inhabit a wide range of sponge hosts around the world [6]. Usher [6] states that it is difficult to understand how the symbionts maintain species identity, given the geographic and reproductive isolation and different habitat; one of the possible explanations she gave was the low resolution of 16S rDNA at the species level, and underlined that the determination of the true number of species represented by cyanobacterial symbionts clades is important for understanding both ecology and evolutionary history of this symbioses. Nevertheless, recent research revealed new cyanobacterial genera in association with sponges; *Anabaena*, *Acaryochloris*, *Cyanobium*, *Halomicronema*, *Myxosarcina*, *Prochloron*, and *Xenococcus* have been found with microscopy, isolation, and cloning techniques (e.g., [59, 65]). Erwin et al. [60] found the cyanobacterium, *Synechocystis trididemni* in *Ircinia fasciculata*, which corresponded to a new symbiont clade, whereas Alex et al. [59] described new sponge-associated cyanobacterial morphotypes (*Xenococcus*-like) in *Hymeniacidon perlevis* and observed *Acaryochloris* sp. for the first time as sponge symbionts. Caroppo et al. [56] characterized a new species, *Halomicronema metazoicum*, isolated from Mediterranean *Petrosia ficiformis* specimens. Sequences retrieved with NGS have been assigned to the cyanobacterial genera *Calothrix*, *Pleurocapsa*, *Chroococcidiopsis*, *Lyngbya*, *Desmonostoc*, *Crocosphaera*, *Mastigocladus*, *Microcoleus*, *Hydrocoleum*, *Phormidium*, and *Pseudanabaena* (e.g. [83, 84]) (S4 Table and references therein). Those results show that the cyanobacterial diversity known from sponges is not yet adequately known.

## Conclusions

The intensification of research using new sequencing technologies has greatly increased the information on bacteria-hosting sponges. On the contrary, and because of these technologies, the taxonomic information below the Phylum level has often received less attention. The meta-analysis performed in this study showed that the reported number of sponge species associated with cyanobacteria has increased by almost 300% in the last 10 years (partly because of an increase in the research effort), but the global cyanobacterial diversity reported from sponges remains underestimated. All poriferan classes can host cyanobacteria, whereas some sponge orders appear to harbour cyanobacteria more frequently than others (Chondrosiida, Chondrillida, Verongiida, Dictyoceratida and Scopanilida). Moreover, the research effort is not equally distributed over the world ocean, with the Tropical Northwestern Atlantic and Mediterranean Sea having received most attention. The eastern basin of Mediterranean Sea which remained almost unexplored, has nearly reached the Western Basin in records of sponge-cyanobacteria associations after this study. Overall, cyanobacterial associations with sponges appear to be as common in temperate areas as in tropical ones.

Our research revealed that sponge-cyanobacteria association seems to be a frequent phenomenon in the Aegean Sea: nine new records of sponge species, previously unknown for this association, were added, and nine isolated cyanobacteria were found to form novel clades. Further investigation is required in order to clarify the taxonomic status of these new strains.

## Supporting information

S1 Appendix Description of isolated strains. (DOCX)

S1 Fig Phylogenetic tree based on 16S rRNA gene sequences of the Leptolyngbyaceae and closely related Pseudanabaenaceae and Heteroleibleiniaceae families, and reconstructed using the Maximum-Likelihood (ML) analysis. Numbers above branches indicate the bootstrap value (as percentages of 1,000 replications) for NJ/ML methods. Strains of the present study are indicated in bold, GenBank accession numbers are indicated in brackets. Bar represents 0.1 nucleotide substitutions per site.
(TIF)

S1 Table Occurrence of sponge-associated cyanobacteria (F: filament morphs, C: coccoid morphs) in sponge specimens from different habitat types and depths of the study site (Chalkidiki Peninsula, North Aegean Sea). Ov = overhang, Rr = rocky reef, Vw = Vertical wall.
(DOCX)

S2 Table PCR primers used in this study. (DOCX)

S3 Table Cyanobacteria strains isolated from sponges. (DOCX)

S4 Table Data set. (XLSX)

S5 Table Cyanobacteria taxa most frequently found in association with different sponge genera. (DOCX)

S1 Text Prisma checklist. (DOCX)

S2 Text Catalogue of literature for S4 Table. (DOCX)
