# Supplementary material for: Sponges-Cyanobacteria associations: Global diversity overview and new data from the Eastern Mediterranean
Source: PLoS One. 2018 Mar 29;13(3):e0195001. doi: 10.1371/journal.pone.0195001 (PMC5875796; doi:10.1371/journal.pone.0195001)
Supplement: S1 Appendix — (DOCX) [file pone.0195001.s001.docx]

# S1 Appendix. Description of Isolated strains

## Strain TAU-MAC 0615

The strain was assigned to the genus *Xenococcus* Thuret, 1880 (Fig 3), but it did not exhibit the morphological features of any of the species [1]; it shared more common morphological traits with the species *Xenococcus pallidus,* however some differences were observed in morphology (smaller cell diameter) and ecology (*X. pallidus* is epiphytic on seaweeds) [1].

Molecular phylogeny places *Xenococcus* TAU-MAC 0615, with very good node support, in a clade with the strain *Xenococcus* PCC 7305, within Pleurocapsales (Fig 4). Strain TAU-MAC 0615 showed 98% pairwise sequence identity with *Xenococcus* PCC 7305 strain, which was isolated from a marine environment [2] and shares some morphological features with our strain. However, *Xenococcus* PCC 7305 has larger cell diameter (maximum 25 μm sensu Komárek and Anagnostidis [1]) compared to our strain. This is the first report of a cyanobacterium symbiont belonging to the genus *Xenococcus* in association with the sponge *Ircinia variabilis.*

## Strains TAU-MAC 0715, 0815

The strains exhibited all the typical morphological features (Fig 3) of the genus *Synechococcus* Nägeli 1849 given in Komárek and Anagnostidis [1]. None of the strains exhibited any morphological traits with known *Synechococcus* species associated with sponges, such as *S. feldmannii* and *S. spongiarum*. The strains TAU-MAC 0715 and 0815 were assigned to the taxon *Synechococcus* sp.

Strains TAU-MAC 0715 and 0815 share 97.5 % identity in 16S RNA gene. The best blastn hit (99-98% similarity) for both 16S rRNA sequences was an uncultured bacterium, derived from corals with White Patch Syndrome in the Pacific Ocean [3]. A 96%-95% similarity was found with a bacterium clone recovered from the sponge *Crella cyathophora* [4]. SILVA taxonomy assigned these uncultured bacteria clones to the genus *Synechococcus*. Our strains and the two bacteria clones clustered together in a distinct clade, well supported by bootstrap values (100%) (Fig 4). This clade was placed outside the free-living *Synechococcus* clades and the sponge-specific clade *Candidatus Synechococcus spongiarum*. *Synechococcus* spp. are the most common cyanobacteria found in sponges and include symbionts affiliated with free-living species and the widespread “*Ca. Synechococcus spongiarum*” [5, 6, 7, 8] which comprises at least 12 different subclusters [9]. Moreover, *Synechococcus* is polyphyletic, it has a global distribution and is poorly defined with respect to its morphology and ecology [10, 11]. The low 16S rRNA gene (<94%) pairwise sequence identity of our strains with other *Synechococcus* strains, as well as their distinct morphology and ecology indicate that these strains could belong to a new species.

## Strains TAU-MAC 0915, 1015, 1115, 1215

The four isolates exhibited all the typical features (Fig 3) of *Leptolyngbya* [12] but none of them had all the morphological traits of any species of the genus. However, our strains shared many morphological traits with *Leptolyngbya ectocarpii*, exhibiting some differences in cell length and width and apical cell formation; *L. ectocarpii* is a marine benthic species, epiphytic on various seaweeds and epizoic on various animals, widely distributed in littoral and sublittoral zones [12]. Phylogenetic analysis placed our strains close to marine *L. ectocarpii* strains (Fig 5). However, strains TAU-MAC 0915, 1015 and 1115 formed a separate subcluster with very high bootstrap values while TAU-MAC 1215 was placed outside this clade (Fig 5). Strains TAU-MAC 0915 and 1015 showed 98% pairwise sequence similarity with *L. ectocarpii* strains, whereas strains TAU-MAC 1115 and 1215 showed 97% similarity. Moreover, our strains share 97% pairwise sequence similarity with other *Leptolyngbya* strains (also close to *L. ectocarpii*), such as strain HBC2 isolated from stromatolites in Bahamas [13], strain RSO2 isolated from the Red Sea [14], and two *Leptolyngbya* clones (FLKB and P2b-2) retrieved from corals with Black Band Disease [15].

A more detailed phylogenetic analysis of Leptolyngbyaceae family (S1 Fig) revealed that our strains clustered within the thin-filament marine genera *Halomicronema*, *Haloleptolyngbya*, and *Nodosilinea* clade and were well separated from other *Leptolyngbya* species (such as *L. crispata, L. corticola, L. tenerrima* and *L. boryana*) isolated from freshwaters. The observed differences in morphology, phylogeny, and ecology suggest that the *Leptolyngbya* strains isolated in this study could belong to a new species.

## Strain TAU-MAC 1415

The strain exhibited many of the typical features of *Pseudanabaena persicina* (Fig 3), and thus it was assigned to the taxon *Pseudanabaena* cf. *persicina*. According to Komárek and Anagnostidis [12], *P. persicina* is a marine cyanobacterium found in association with sponges (based on culture-independent techniques).

Our strain showed 98% pairwise sequence similarity and clustered together in a distinct clade with the strain Cf *Symploca* sp. FK07-1 [16] (Fig 5). Morphological description of the strain Cf *Symploca* sp. FK07-1 is not available, but Salvador-Reyes et al. [16] indicate that corresponds to traditional taxonomic definition of *Symploca* spp.; phylogenetic analysis has shown that this strain is part of a separate and distinct lineage, distantly related to any known taxa [16]. In our analysis, the two strains (TAU-MAC 1415 and Cf *Symploca* sp. FK07-1) and the strain *Leptolyngbya* sp. RSO1 formed a separate clade outside other *Symploca* and *Pseudanabaena* strains (Fig 5 and S1 Fig) and within Leptolyngbyaceae (S1 Fig)*.* These observations could be explained by the fact that the families Leptolyngbyaceae and Pseudanabaenaceae are polyphyletic and closely related [11]. Our results suggest that strains TAU-MAC 1415, Cf *Symploca* sp. FK07-1 and *Leptolyngbya* sp. RSO1 are part of a new lineage.

## Strain TAU-MAC 1315

Strain TAU-MAC 1315 exhibited the typical morphological features of the family Schizotrichaceae (Fig 3), described in Komárek and Anagnostidis [12]. The strain did not exhibit the typical features of any genus in the family, although it shared some traits with the genus *Schizothrix.* Since it could not be assigned to a taxon at the genus level, it was denoted as Schizotrichaceae sp.

Our strain formed a separated subcluster in a well-supported clade (100% bootstrap value), which included the strains *Leptolyngbya* sp. ANT.L52.1 and *Pseudophormidium* sp./*Schizothrix* sp. ANT.LPE isolated from Antarctic lakes [17] (Fig 5). This clade shows significant divergence from the strains of the family Schizotrichaceae and seems to be more closely related to the marine *Leptolyngbya* strains and other thin filamentous Leptolyngbyaceae (i.e. *Halomicronema, Nodosilinea, Ocullatela, Haloleptolyngbya*) (S1 Fig). Schizotrichaceae family lacks molecular data; only five *Schizothrix* sequences exist, and were not all included in our phylogenetic analysis due to their short length (<900 bases). Strain TAU-MAC 1315 showed <96% pairwise sequence identity with both Antarctic strains. According to Komárek [18] similarity of ±95% or less in 16S rRNA gene sequences, means that possibly is a different genus. Both morphology and phylogeny suggest that our strain cannot be classified into any of the existing genera and might represent a new taxon at the genus level.

# References

1. Komárek J, Anagnostidis K. Cyanoprokaryota 1. Teil: Chroococcales. In: Ettl H, Gärtner G, Heynig H, Mollenhauer D, editors. Süßwasserflora von Mitteleuropa 19/1. Stuttgart: Gustav Fischer; 1999.

2. Waterbury JB, Stanier RY. Patterns of growth and development in Pleurocapsalean Cyanobacteria. Microbiol Rev. 1978;42: 2-44.

3. Séré MG, Tortosa P, Chabanet P, Turquet J, Quod JP, Schleyer MH. Bacterial communities associated with *Porites* White Patch Syndrome (PWPS) on three Western Indian Ocean (WIO) coral reefs. Plos ONE. 2013;8: e83746.

4.Giles EC, Kamke J, Moitinho-Silva L, Taylor MW, Hentschel U, Ravasi T, et al. Bacterial community profiles in low microbial abundance sponges. FEMS Microbiol Ecol. 2013;83: 232-241.

5. Usher KM. The ecology and phylogeny of cyanobacterial symbionts in sponges. Mar Ecol. 2008;29: 178-192.

6. Steindler L, Huchon D, Avni A, Ilan M. 16S rRNA phylogeny of sponge-associated cyanobacteria. Appl Environ Microb. 2005;71: 4127-4131.

7. Gao ZM, Wang Y, Tian RM, Wong YH, Batang ZB, Al-Suwailem AM, et al. Symbiotic adaptation drives genome streamlining of the cyanobacterial sponge symbiont “*Candidatus* *Synechococcus spongiarum*”. MBio. 2014;5: e00079-14.

8. Burgsdorf I, Slaby BM, Handley KM, Haber M, Blom, J, Marshall CW, et al. Lifestyle evolution in cyanobacterial symbionts of sponges. MBio. 2015;6: e00391-15.

9. Erwin PM, Thacker RW. Cryptic diversity of the symbiotic cyanobacterium *Synechococcus spongiarum* among sponge hosts. Mol Ecol. 2008a;17: 2937-2947.

10. Robertson BR, Tezuka N, Watanabe MM. Phylogenetic analyses of *Synechococcus* strains (cyanobacteria) using sequences of 16S rDNA and part of the phycocyanin operon reveal multiple evolutionary lines and reflect, phycobilin content. Int J Syst Evol Micr. 2001;51: 861-871.

11. Komárek J, Kaštovský J, Mareš J, Johansen JR. Taxonomic classification of cyanoprokaryotes (cyanobacterial genera) 2014, using a polyphasic approach. Preslia. 2014;86: 295-335.

12. Komárek J, Anagnostidis K. Cyanoprokaryota 2. Teil: Oscillatoriales. In: Büdel B, Gärtner G, Krienitz L, Schagerl M, editors. Süßwasserflora von Mitteleuropa 19/2. München: Elsevier Spektrum Akademischer Verlag; 2005.

13. Foster JS, Green SJ, Ahrendt SR, Golubic S, Reid RP, Hetherington L, et al. Molecular and morphological characterization of cyanobacterial diversity in the stromatolites of Highborne Cay, Bahamas. ISME J. 2009;3: 573-587

14. Thornburg CC, Thimmaiah M, Shaala LA, Hau AM, Malmo JM, Ishmael JE, et al. Cyclic Depsipeptides, Grassypeptolides D and E and Ibu-epidemethoxylyngbyastatin 3, from a Red Sea *Leptolyngbya* cyanobacterium. J Nat Prod. 2011;74: 1677-1685.

15. Myers JL, Sekar R, Richardson LL. Molecular detection and ecological significance of the cyanobacterial genera *Geitlerinema* and *Leptolyngbya* in Black Band Disease of Corals. Appl Environ Microbiol. 2007;73: 5173-5182.

16. Salvador-Reyes LA, Engene N, Paul VJ, Luesch H Targeted natural products discovery from marine cyanobacteria using combined phylogenetic and mass spectrometric evaluation. J Nat Prod. 2015;78: 486-492.

17. Taton A, Grubisic S, Ertz D, Hodgson DA, Picardi R, Biondi N, et al. Polyphasic study of Antarctic cyanobacterial strains. J Phycol. 2006;42: 1257-1270.

18. Komárek J. Recent changes (2008) in cyanobacterial taxonomy based on a combination of molecular background with phenotype and ecological consequences (genus and species concept). Hydrobiologia. 2010;639: 245-259
