# Supplementary figures and images for: Sponges-Cyanobacteria associations: Global diversity overview and new data from the Eastern Mediterranean
Source: PLoS One. 2018 Mar 29;13(3):e0195001. doi: 10.1371/journal.pone.0195001 (PMC5875796; doi:10.1371/journal.pone.0195001)

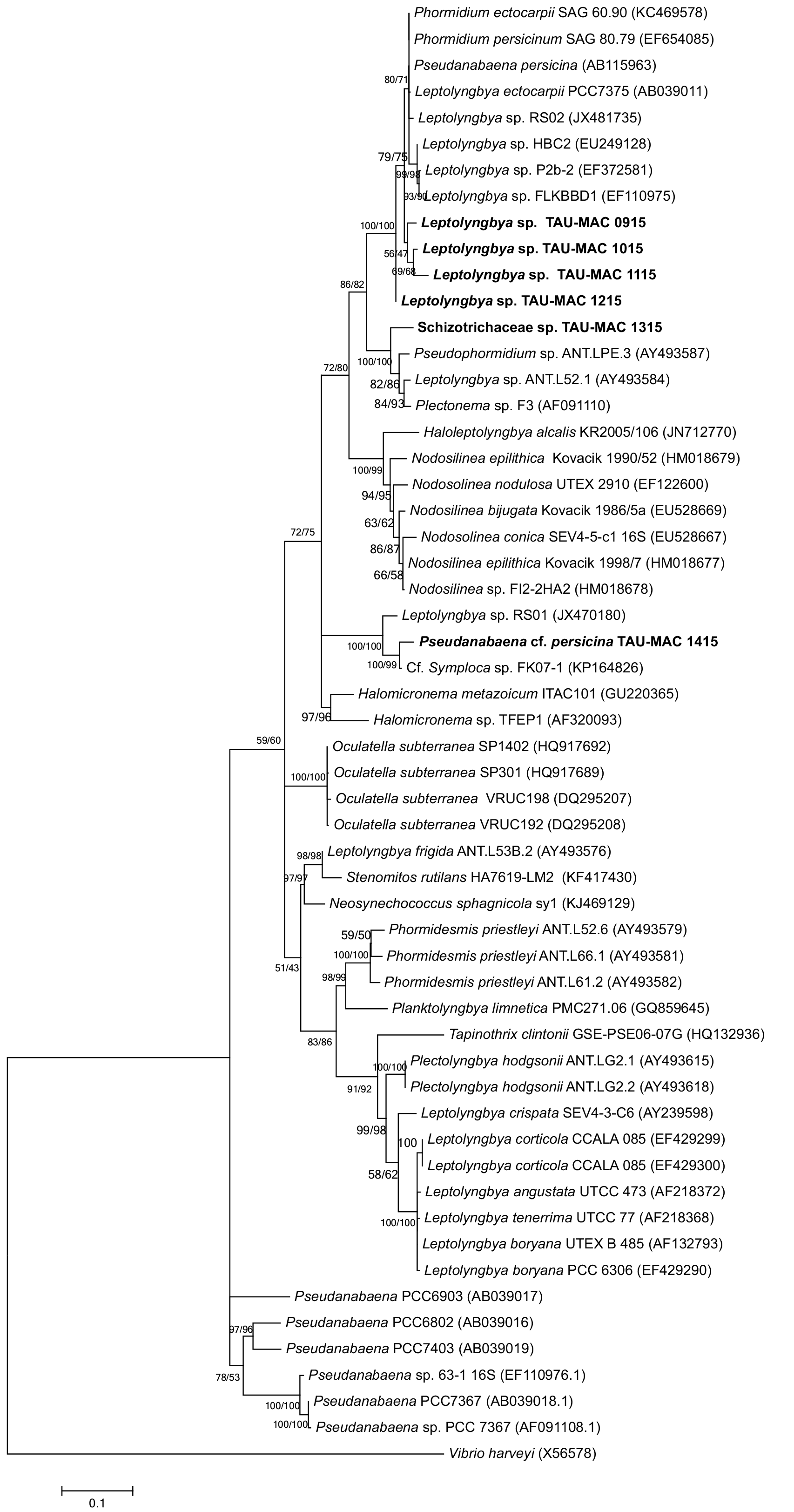

Supplement: S1 Fig — Numbers above branches indicate the bootstrap value (as percentages of 1,000 replications) for NJ/ML methods. Strains of the present study are indicated in bold, GenBank accession numbers are indicated in brackets. Bar represents 0.1 nucleotide substitutions per site. (TIF) [file pone.0195001.s002.tif]
