# Supplementary material for: Sponges-Cyanobacteria associations: Global diversity overview and new data from the Eastern Mediterranean
Source: PLoS One. 2018 Mar 29;13(3):e0195001. doi: 10.1371/journal.pone.0195001 (PMC5875796; doi:10.1371/journal.pone.0195001)
Supplement: S1 Table — Ov = overhang, Rr = rocky reef, Vw = Vertical wall. (DOCX) [file pone.0195001.s003.docx]

**S1** **Table.** Occurrence of sponge-associated cyanobacteria (F: filament morphs, C: coccoid morphs) in sponge specimens from different habitat types and depths of the study site (Chalkidiki Peninsula, North Aegean Sea); Ov=overhang, Rr=rocky reef, Vw=Vertical wall.

| **Specimen** | **Sponge species** | **Habitat type**  **(depth in m)** | **Cyanobacteria** | | | |
| --- | --- | --- | --- | --- | --- | --- |
|  |  |  | **PCR screening** | **Autofluorescent signal** | **Morphotype** | **Isolate** |
| 1 | *Acanthella acuta* Schmidt, 1862 | Ov (16.7) | + | + | C, F | *Leptolyngbya* sp. TAU-MAC 1115 |
|  | *Agelas oroides* (Schmidt, 1864) |  |  |  |  | - |
| 2 | *A.o*.1 – massive specimen | Rr (9.6) | - | + | F | - |
| 3 | *A.o*.2 – massive specimen | Vw (16.7) | - | + | C | - |
| 4 | *A.o*.3 – massive-tubular specimen | Ov (16.7) | + | + | C | - |
|  | *Aplysina aerophoba* (Nardo, 1833) |  |  |  |  | - |
| 5 | *A.a*.1 – sample 1 | Rr (6.8) | - | + | C | - |
| 6 | *A.a.*2 – sample 2 | Rr (6.8) | + | + | C | Schizotrichaceae sp. TAU-MAC 1315 |
| 7 | *Axinella cannabina* (Esper, 1794) | Ov (16.7) | + | + | C | *Synechococcus* sp. TAU-MAC 0715 |
| 8 | *Axinella damicornis* (Esper, 1794) | Ov (21) | + | + | C | *Synechococcus* sp. TAU-MAC 0815,  *Pseudanabaena* cf. *persicina* TAU-MAC 1415 |
| 9 | *Axinella verrucosa* (Esper, 1794) | Ov (16.7) | - | + | C | - |
| 10 | *Chondrilla nucula* Schmidt, 1862 | Rr (9.6) | + | + | C | *Leptolyngbya* sp. TAU-MAC 1215 |
|  | *Chondrosia reniformis* Nardo, 1847 |  |  |  |  | - |
| 11 | *C.r.*1 – sample 1 | Rr (9.6) | - | - | - | - |
| 12 | *C.r.*2 – sample 2 | Ov (16.7) | + | - | - | - |
| 13 | *Dysidea avara* (Schmidt, 1862) | Rr (19.5) | - | + | C | *Leptolyngbya* sp. TAU-MAC 1015 |
| 14 | *Haliclona (Halichoclona) fulva* (Topsent, 1893) | Ov (21) | - | + | C | - |
|  | *Hexadella racovitzai* Topsent, 1896 |  |  |  |  | - |
| 16 | *H.r.*1 –faded pink colour form | Ov (21) | + | + | C | - |
| 17 | *H.r.*2 –pale pink colour form | Ov (21) | - | + | C | - |
| 18 | *Ircinia oros* (Schmidt, 1864) | Rr (9.6) | - | - | - | - |
| 19 | *Ircinia variabilis* (Schmidt, 1862) | Rr (9.8) | + | + | C | *Xenococcus* sp. TAU-MAC 0615 |
| 20 | *Oscarella* sp. | Ov (19.4) | - | + | C |  |
| 21 | *Petrosia (Petrosia) ficiformis* (Poiret, 1789) | Rr (19.5) | - | + | C | *Leptolyngbya* sp. TAU-MAC 0915 |
| 22 | *Sarcotragus foetidus* Schmidt, 1862 | Rr (9.8) | - | - | - | - |
|  | *Spirastrella cunctatrix* Schmidt, 1868 |  |  |  |  | - |
| 23 | *S.c.*1 – sample 1 | Rr (9.6) | + | + | C | - |
| 24 | *S.c.*2 – sample 2 | Vw (19.4) | + | + | C, F | - |
| 25 | *Stryphnus ponderosus* (Bowerbank, 1866) | Rr (19.3) | + | + | C | - |
