# Supplementary material for: Sponges-Cyanobacteria associations: Global diversity overview and new data from the Eastern Mediterranean
Source: PLoS One. 2018 Mar 29;13(3):e0195001. doi: 10.1371/journal.pone.0195001 (PMC5875796; doi:10.1371/journal.pone.0195001)
Supplement: S2 Table — (DOCX) [file pone.0195001.s004.docx]

**S2** **Table.** PCR primers used in this study.

| **Primers^α^** | **Sequence (5’-3’)** | **Reference** |
| --- | --- | --- |
| CYA106F | CGGACGGGTGAGTAACGCGTGA | Nübel et al. 1997 |
| 16S27F | AGAGTTTGATCTGGCTC G | Taton et al. 2003 |
| 23S30R | CTTCGCCTCTGTGTGCCTAGGT | Taton et al. 2003 |
| CYA781R(a) | GACTACTGGGGTATCTAATCCCATT | Nübel et al. 1997 |
| CYA781R(b) | GACTACAGGGGTATCTAATCCCTTT | Nübel et al. 1997 |
