# Supplementary material for: Sponges-Cyanobacteria associations: Global diversity overview and new data from the Eastern Mediterranean
Source: PLoS One. 2018 Mar 29;13(3):e0195001. doi: 10.1371/journal.pone.0195001 (PMC5875796; doi:10.1371/journal.pone.0195001)
Supplement: S3 Table — (DOCX) [file pone.0195001.s005.docx]

**S3** **Table.** Cyanobacteria strains isolated from sponges.

| **Cyanobacteria strains** | **Sponge host** | **Reference** |
| --- | --- | --- |
| *Cyanobium* sp. | *Petrosia* (*Petrosia*) *ficiformis* | Pagliara and Caroppo, 2011 |
| *Halomicronema metazoicum* | *Petrosia* (*Petrosia*) *ficiformis* | Caroppo et al. 2012 |
| *Leptolyngbya* cfr. *ectocarpii* | *Petrosia* (*Petrosia*) *ficiformis* | Pagliara and Caroppo, 2011 |
| *Leptolyngbya* cfr. *minuta* | *Petrosia* (*Petrosia*) *ficiformis* | Pagliara and Caroppo, 2011 |
| *Leptolyngbya* sp. | *Petrosia* (*Petrosia*) *ficiformis* | Pagliara and Caroppo, 2011 |
| *Leptolyngbya* sp. | *Aplysina cauliformis* | Olson et al. 2014 |
| *Leptolyngbya* or *Plectonema*-like | *Rhopaloeides odorabile* | Webster and Hill, 2001 |
| *Myxosarcina* sp. | *Terpios hoshinota* | Yu et al. 2015 |
| *Synechococcus* sp. (2 strains) | *Petrosia* (*Petrosia*) *ficiformis* | Pagliara and Caroppo, 2011 |
| 9 unidentified cyanobacteria | *Candidaspongia flabellata* | Burja and Hill, 2001 |
| *Xenococcus* sp. TAU-MAC 0615 | *Ircinia variabilis* | this study |
| *Synechococcus* sp. TAU-MAC 0715 | *Axinella cannabina* | this study |
| *Synechococcus* sp. TAU-MAC 0815 | *Axinella damicornis* | this study |
| *Leptolyngbya* sp. TAU-MAC 0915 | *Petrosia (Petrosia) ficiformis* | this study |
| *Leptolyngbya* sp. TAU-MAC 1015 | *Dysidea avara* | this study |
| *Leptolyngbya* sp. TAU-MAC 1115 | *Acanthella acuta* | this study |
| *Leptolyngbya* sp. TAU-MAC 1215 | *Chondrilla nucula* | this study |
| Schizotrichaceae sp. TAU-MAC 1315 | *Aplysina aerophoba* | this study |
| *Pseudanabaena* cf. *persicina* TAU-MAC 1415 | *Axinella damicornis* | this study |
