# Supplementary material for: Sponges-Cyanobacteria associations: Global diversity overview and new data from the Eastern Mediterranean
Source: PLoS One. 2018 Mar 29;13(3):e0195001. doi: 10.1371/journal.pone.0195001 (PMC5875796; doi:10.1371/journal.pone.0195001)
Supplement: S5 Table — (DOCX) [file pone.0195001.s007.docx]

**Table S5.** **Cyanobacteria taxa most frequently found in association with different sponge genera.**

| **Taxon** | **Sponge genera** | **Representative References*** |
| --- | --- | --- |
| *Ca*ndidatus *Synechococcus spongiarum* | *Aplysina, Carteriospongia, Chondrilla, Cinachyrella, Coelocarteria, Coscinoderma, Cymbastela, Diacarnus, Haliclona, Hamigera, Hippospongia, Ianthella, Ircinia, Neopetrosia, Petrosia, Phyllospongia, Psammastra, Pseudoceratina, Rhopaloeides, Sarcotragus, Smenospongia, Spheciospongia, Stelletta, Stylissa, Svenzea, Tedania, Terpios, Theonella, Verongula, Xestospongia* | [21, 22, 23, 52, 77, 89,90, 93, 94, 97, 98, 105] |
| *Synechococcus* spp. | *Acanthella, Agelas, Aplysina, Astrosclera, Axinella, Callyspongia, Calyx, Carteriospongia, Chondrilla, Clathria,* *Crambe, Craniella,* *Cribrochalina,* *Cliona, Coscinoderma, Crella, Darwinella, Dendrilla, Discodermia, Dragmacidon, Dysidea, Gelliodes, Halichondria, Haliclona, Hymeniacidon, Ianthella, Ircinia, Lissodendoryx, Mycale, Neamphius, Neofibularia, Neopetrosia, Oscarella, Penares, Pericharax, Petrosia,* *Phyllospongia*, *Psammocinia, Rhabdastrella, Rhabderemia, Rhaphoxya, Siphonochalina, Suberites, Tethya, Trachytedania, Xestospongia* | [3, 27, 36, 34, 41, 44, 47, 51, 52, 57, 61, 66, 69, 78, 79, 84, 89, 90,93, 99, 108] |
| *Prochlorococcus* spp. | *Acanthella, Amphilectus, Aplysilla, Aplysina, Axinella, Callyspongia, Dragmacidon, Halichondria, Haliclona, Leucetta, Ophlitaspongia, Phorbas, Polymastia, Rhaphoxya, Suberites* | [2, 28, 59, 66, 78, 99, 108] |
| *Aphanocapsa raspaigella*-like | *Amphimedon, Aplysilla, Clathrina, Dictyonella, Halichondria, Haliclona, Ircinia, Lendenfeldia, Oceanapia, Phorbas, Sycon* | [17, 71, 74,100, 102 ] |
| *Synechocystis* spp. | *Axinyssa, Batzella, Ircinia, Lendenfeldia, Mycale, Phorbas, Sarcotragus, Spirastrella, Spongia, Xestospongia* | [14, 23, 47, 52, 70, 95] |
| *Oscillatoria spongeliae* | *Chondropsis, Dysidea, Haliclona, Hyrtios, Lamellodysidea, Lendenfeldia, Leucetta, Niphates, Phyllospongia, Suberea, Tethya, Theonella, Xestospongia* | [17, 28, 49, 52, 70, 83] |
| *Leptolyngbya* spp. | *Aplysina, Carteriospongia, Ircinia, Leucetta, Mycale, Petrosia, Rhopaloeides* | [28, 30, 59, 63, 96, 107] |
| Cyanobacteria (records of presence) | *Ancorina,* *Aiolochroia, Aphrocallistes, Arenosclera, Asteropus, Biemna, Candidaspongia, Caulospongia, Ceratoporella,* *Chalinula,* *Chelonaplysilla,* *Ciocalypta,* *Chondrosia, Dactylospongia, Echinodictyum,* *Ecionemia, Fibulia,* *Iotrochota, Leiodermatium, Luffariella, Monanchora,* *Myxilla, Paratetilla, Phakellia, Pheronema,* *Plakina,* *Plakortis,* *Pseudocorticium, Raspailia, Scopalina, Vaceletia* | [6, 19, 35, 44, 47, 75, 83, 85] |

*The numbered literature is given in S2 Text
