# Supplementary material for: Sponges-Cyanobacteria associations: Global diversity overview and new data from the Eastern Mediterranean
Source: PLoS One. 2018 Mar 29;13(3):e0195001. doi: 10.1371/journal.pone.0195001 (PMC5875796; doi:10.1371/journal.pone.0195001)
Supplement: S2 Text — (DOCX) [file pone.0195001.s009.docx]

S2 Text. Catalogue of Literature for S4 Table

1. Abe T, Sahin FP, Akiyama K, Naito T, Kishigami M, Miyamoto K, et al. Construction of a metagenomic library for the marine sponge *Halichondria okadai*. Biosci Biotech Bioch. 2012;76: 633-639.

2. Alex A, Antunes A. Pyrosequencing characterization of the microbiota from Atlantic intertidal marine sponges reveals high microbial diversity and the lack of co-occurrence patterns. PloS ONE. 2015;10: e0127455.

3. Alex A, Vasconcelos V, Tamagnini P, Santos A, Antunes A. Unusual symbiotic cyanobacteria association in the genetically diverse intertidal marine sponge *Hymeniacidon perlevis* (Demospongiae, Halichondrida). PLoS ONE. 2012;7: e51834.

4. Angermeier H, Kamke J, Abdelmohsen UR, Krohne G, Pawlik JR, Lindquist NL, et al. The pathology of sponge orange band disease affecting the Caribbean barrel sponge *Xestospongia muta*. FEMS Microbiol Ecol. 2011;75: 218-230.

5. Arillo A, Bavestrello G, Burlando B, Sarà M. Metabolic integration between symbiotic cyanobacteria and sponges-a possible mechanism. Mar Biol. 1993;117: 159-162.

6. Bayer K, Kamke J, Hentschel U. Quantification of bacterial and archaeal symbionts in high and low microbial abundance sponges using real-time PCR. FEMS Microbiol Ecol. 2014;89: 679-690.

7. Buccella C, Alvarez B, Gibb K, Padovan A. A rod-like bacterium is responsible for high molybdenum concentrations in the tropical sponge *Halichondria phakellioides*. Mar Freshwater Res. 2014;65: 838-848.

8. Burgsdorf I, Erwin PM, López-Legentil S, Cerrano C, Haber M, Frenk S, et al. Biogeography rather than association with cyanobacteria structures symbiotic microbial communities in the marine sponge *Petrosia ficiformis*. Front Microbiol. 2014;5: 529.

9. Burgsdorf I, Slaby BM, Handley KM, Haber M, Blom, J, Marshall CW, et al. Lifestyle evolution in cyanobacterial symbionts of sponges. MBio. 2015;6: e00391-15.

10. Burja AM, Hill RT. Microbial symbionts of the Australian Great Barrier reef sponge, *Candidaspongia flabellata*. Hydrobiologia. 2001;461: 41-47.

11. Cao H, Cao X, Guan X, Xue S, Zhang W. High temporal variability in bacterial community, silicatein and hsp70 expression during the annual life cycle of *Hymeniacidon sinapium* (Demospongiae) in China's Yellow Sea. Aquaculture. 2012;358: 262-273.

12. Cárdenas CA, Bell JJ, Davy SK, Hoggard M, Taylor MW. Influence of environmental variation on symbiotic bacterial communities of two temperate sponges. FEMS Microbiol Ecol. 2014;88: 516-527.

13. Caroppo C, Albertano P, Bruno L, Montinari M, Rizzi M, Vigliotta G, et al. Identification and characterization of a new *Halomicronema* species (Cyanobacteria) isolated from the Mediterranean marine sponge *Petrosia ficiformis* (Porifera). Fottea Olomouc. 2012;12: 315-326.

14. Cox GC, Hiller RG, Larkum AWD. An unusual cyanophyte, containing phycourobilin and symbiotic with ascidians and sponges. Mar Biol. 1985;89: 149-163.

15. Cuvelier ML, Blake E, Mulheron R, McCarthy PJ, Blackwelder P, Thurber RLV, et al. Two distinct microbial communities revealed in the sponge *Cinachyrella*. Front Microbiol. 2014;5: 581.

16. de Voogd NJ, Cleary DF, Polónia AR, Gomes NC. Bacterial community composition and predicted functional ecology of sponges, sediment and seawater from the thousand islands reef complex, West Java, Indonesia. FEMS Microbiol Ecol. 2015;91: fiv019.

17. Diaz MC, Thacker RW, Rützler K, Piantoni C. Two new haplosclerid sponges from Caribbean Panama with symbiotic filamentous cyanobacteria, and an overview of sponge-cyanobacteria associations. In: Custódio MR, Lôbo-Hajdu G, Hajdu E, Muricy G, editors. Porifera Research: Biodiversity, Innovation and Sustainability. Rio de Janeiro: Museu Nacional; 2007. pp. 31-39.

18. Drozdov AL, Bukin OA, Voznesenskii SS, Galkina AN, Golik SS, Zhukova NV, et al. Symbiont cyanobacteria in the Hexactinellid sponges (Porifera: Hexactinellida). Dokl Biol Sci. 2008;420: 192-194.

19. Easson CG, Thacker RW. Phylogenetic signal in the community structure of host-specific microbiomes of tropical marine sponges. Front Microbiol. 2014;5: 532.

20. Erwin PM, Thacker RW. Incidence and identity of photosynthetic symbionts in Caribbean coral reef sponge assemblages. J Mar Biol Assoc UK. 2007;87: 1683-1692.

21. Erwin PM, Thacker RW. Cryptic diversity of the symbiotic cyanobacterium *Synechococcus spongiarum* among sponge hosts. Mol Ecol. 2008a;17: 2937-2947.

22. Erwin PM, Thacker RW. Phototrophic nutrition and symbiont diversity of two Caribbean sponge-cyanobacteria symbioses. Mar Ecol Prog Ser. 2008b;362: 139-147.

23. Erwin PM, López-Legentil S, Turon X. Ultrastructure, molecular phylogenetics, and chlorophyll a content of novel cyanobacterial symbionts in temperate sponges. Microb Ecol. 2012;64: 771-783.

24. Fan L, Reynolds D, Liu M, Stark M, Kjelleberg S, Webster NS, et al. (2012) Functional equivalence and evolutionary convergence in complex communities of microbial sponge symbionts. Proc Natl Acad Sci USA. 2012;109: E1878–E1887.

25. Fiore CL, Jarett JK, Lesser MP. Symbiotic prokaryotic communities from different populations of the giant barrel sponge, *Xestospongia muta*. Microbiologyopen. 2013;2: 938-952.

26. Freeman CJ, Thacker RW. Complex interactions between marine sponges and their symbiotic microbial communities. Limnol Oceanogr. 2011;56: 1577-1586.

27. Freckelton ML, Luter HM, Andreakis N, Webster NS, Motti CA. Qualitative variation in colour morphotypes of *Ianthella basta* (Porifera: Verongida). Hydrobiologia. 2012;687: 191-203.

28. Fromont J, Huggett MJ, Lengger SK, Grice K, Schönberg CH. Characterization of *Leucetta prolifera*, a calcarean cyanosponge from south-western Australia, and its symbionts. J Mar Biol Assoc UK. 2016;96: 541-552.

29. Gaino E, Sciscioli M, Lepore E, Rebora M, Corriero G. Association of the sponge *Tethya orphei* (Porifera, Demospongiae) with filamentous cyanobacteria. Invertebr Biol. 2006;12: 281-287.

30. Gao ZM, Wang Y, Lee OO, Tian RM, Wong YH, Bougouffa S, et al. Pyrosequencing reveals the microbial communities in the Red Sea sponge Carteriospongia foliascens and their impressive shifts in abnormal tissues. Microb Ecol. 2014;68: 621-632.

31. Gao ZM, Wang Y, Tian RM, Lee OO, Wong YH, Batang ZB, et al. Pyrosequencing revealed shifts of prokaryotic communities between healthy and disease-like tissues of the Red Sea sponge *Crella cyathophora*. PeerJ. 2015;3: e890.

32. Gerçe B, Schwartz T, Voigt M, Rühle S, Kirchen S, Putz A, et al. Morphological, bacterial, and secondary metabolite changes of *Aplysina aerophoba* upon long-term maintenance under artificial conditions. Microb Ecol. 2009;58: 865-878.

33. Giles EC, Kamke J, Moitinho-Silva L, Taylor MW, Hentschel U, Ravasi T, et al. (2013) Bacterial community profiles in low microbial abundance sponges. FEMS Microbiol Ecol. 2009; 83:232-241.

34. Gloeckner V, Hentschel U, Ereskovsky AV, Schmitt S. Unique and species-specific microbial communities in *Oscarella lobularis* and other Mediterranean Oscarella species (Porifera: Homoscleromorpha). Mar Biol. 2013;160: 781-791.

35. Gloeckner V, Wehrl M, Moitinho-Silva L, Gernert C, Schupp P, Pawlik J, et al. The HMA-LMA dichotomy revisited: an electron microscopical survey of 56 sponge species. Biol Bull. 2014;227: 78-88.

36. Hardoim CCP, Costa R, Araujo FV, Hajdu E, Peixoto R, Lins U, et al. Diversity of bacteria in the marine sponge *Aplysina fulva* in Brazilian coastal waters. Appl Environ Microbiol. 2009;75: 3331-3343.

37. Hentschel U, Schmid M, Wagner M, Fieseler L, Gernert C, Hacker J. Isolation and phylogenetic analysis of bacteria with antimicrobial activities from the Mediterranean sponges *Aplysina aerophoba* and *Aplysina cavernicola*. FEMS Microbiol Ecol. 2001;35: 305-312.

38. Hill M, Hill A, Lopez N, Harriott O. Sponge-specific bacterial symbionts in the Caribbean sponge, *Chondrilla nucula* (Demospongiae, Chondrosida). Mar Biol. 2006;148: 1221-1230.

39. Hinde R, Pironet F, Borowitzka MA. Isolation of *Oscillatoria spongeliae*, the filamentous cyanobacterial symbiont of the marine sponge *Dysidea herbacea*. Mar Biol. 1994;119: 99-104.

40. Hirose E, Murakami A. Microscopic anatomy and pigment characterization of coral-encrusting black sponge with cyanobacterial symbiont, *Terpios hoshinota*. Zool Sci. 2011;28 :199-205.

41. Isaacs LT, Kan J, Nguyen L, Videau P, Anderson MA, Wright TL, et al. Comparison of the bacterial communities of wild and captive sponge *Clathria prolifera* from the Chesapeake Bay. Mar Biotechnol. 2009;11: 758-770.

42. Jasmin C, Anas A, Nair S. Bacterial diversity associated with *Cinachyra cavernosa* and *Haliclona pigmentifera*, cohabiting sponges in the coral reef ecosystem of Gulf of Mannar, Southeast coast of India. PloS ONE. 2015;10: e0123222.

43. Jeong IH, Park JS. Bacterial Diversity of the South Pacific Sponge, *Dactylospongia metachromia* Based on DGGE Fingerprinting. Kor J Microbiol. 2013;49: 377-382.

44. Karlińska-Batres K, Wörheide G. Microbial diversity in the coralline sponge *Vaceletia crypta*. A Van Leeuw J Microb. 2013a;103: 1041-1056.

45. Karlińska-Batres K, Wörheide G. Phylogenetic diversity and community structure of the symbionts associated with the coralline sponge *Astrosclera willeyana* of the Great Barrier Reef. Microb Ecol. 2013b;65: 740-752.

46. Karlińska-Batres K, Woerheide G. Spatial variability of microbial communities of the coralline demosponge *Astrosclera willeyana* across the Indo-Pacific. Aquat Microb Ecol. 2015;74: 143-156.

47. Keesing JK, Usher KM, Fromont J. First record of photosynthetic cyanobacterial symbionts from mesophotic temperate sponges. Mar Freshwater Res. 2012;63: 403-408.

48. Kennedy J, Codling CE, Jones BV, Dobson AD, Marchesi JR Diversity of microbes associated with the marine sponge, *Haliclona simulans*, isolated from Irish waters and identification of polyketide synthase genes from the sponge metagenome. Environ Microbiol. 2008;10: 1888-1902.

49. Larkum AWD, Cox GC, Hiller RG, Parry DL, Dibbayawan TP. Filamentous cyanophytes containing phycourobilin and in symbiosis with sponges and an ascidian of coral reefs. Mar Biol. 1987; 95: 1-13.

50. Lee OO, Wang Y, Yang J, Lafi FF, Al-Suwailem A, Qian P-Y. Pyrosequencing reveals highly diverse and species-specific microbial communities in sponges from the Red Sea. ISME J. 2011;5: 650-664.

51. Li CQ, Liu WC, Zhu P, Yang JL, Cheng KD. Phylogenetic diversity of bacteria associated with the marine sponge *Gelliodes carnosa* collected from the Hainan Island coastal waters of the South China Sea. Microb Ecol. 2011;62: 800.

52. Lemloh ML, Fromont J, Brümmer F, Usher KM. Diversity and abundance of photosynthetic sponges in temperate Western Australia. BMC Ecol. 2009;9: 4.

53. Luter HM, Widder S, Botté ES, Wahab MA, Whalan S, Moitinho-Silva L, et al. Biogeographic variation in the microbiome of the ecologically important sponge, Carteriospongia foliascens. PeerJ. 2015;3: e1435.

54. Maldonado M. Intergenerational transmission of symbiotic bacteria in oviparous and viviparous demosponges, with emphasis on intracytoplasmically-compartmented bacterial types. J Mar Biol Assoc UK. 2007;87: 1701-1713.

55. Montalvo NF, Hill RT. Sponge-associated bacteria are strictly maintained in two closely related but geographically distant sponge hosts. Appl Environ Microb. 2011;77: 7207-7216.

56. Nozawa Y, Huang YS, Hirose E. Seasonality and lunar periodicity in the sexual reproduction of the coral-killing sponge, *Terpios hoshinota*. Coral Reefs. 2016;35: 1071-1081.

57. Ohkubo S, Miyashita H. Selective detection and phylogenetic diversity of *Acaryochloris* spp. that exist in association with didemnid ascidians and sponge. Microbes Environ. 2012;27: 217-225.

58. Olson JB, Gao X. Characterizing the bacterial associates of three Caribbean sponges along a gradient from shallow to mesophotic depths. FEMS Microbiol Ecol. 2013;85: 74-84.

59. Olson JB, Thacker RW, Gochfeld D. Molecular community profiling reveals impacts of time, space, and disease status on the bacterial community associated with the Caribbean sponge *Aplysina cauliformis*. FEMS Microbiol Ecol. 2014;87: 268-279.

60. Oren M, Steindler L, Ilan M. Transmission, plasticity and the molecular identification of cyanobacterial symbionts in the Red Sea sponge *Diacarnus erythraenus*. Mar Biol. 2005;148: 35-41.

61. Ouyang Y, Dai S, Xie L, Kumar MR, Sun W, Sun H, et al. Isolation of high molecular weight DNA from marine sponge bacteria for BAC library construction. Mar Biotechnol. 2010;12: 318-325.

62. Pfannkuchen M, Schlesinger S, Fels A, Brümmer F. Microscopical techniques reveal the *in situ* microbial association inside *Aplysina aerophoba*, Nardo 1886 (Porifera, Demospongiae, Verongida) almost exclusively consists of cyanobacteria. J Exp Mar Biol Ecol. 2010;390: 169-178.

63. Pagliara P, Caroppo C. Cytotoxic and antimitotic activities in aqueous extracts of eight cyanobacterial strains isolated from the marine sponge *Petrosia ficiformis*. Toxicon. 2011;57: 889-896.

64. Park JS. Bacterial community diversity associated with two marine sponges from the South Pacific Ocean based on 16S rDNA-DGGE analysis. Kor J Microbiol. 2010;46: 255-261.

65. Park JS. Bacterial diversity of the Marine Sponge, *Halichondria panicea* by ARDRA and DGGE. Kor J Microbiol. 2015;51: 398-406.

66. Passarini MR, Miqueletto PB, de Oliveira VM, Sette LD. Molecular diversity of fungal and bacterial communities in the marine sponge *Dragmacidon reticulatum*. J Basic Microb. 2015;55:207-220.

67. Pita L, López-Legentil S, Erwin PM. Biogeography and host fidelity of bacterial communities in Ircinia spp. from the Bahamas. Microb Ecol. 2013a;66: 437-447.

68. Pita L, Turon X, López-Legentil S, Erwin PM. Host rules: spatial stability of bacterial communities associated with marine sponges (*Ircinia* spp.) in the Western Mediterranean Sea. FEMS Microbiol Ecol. 2013b;86: 268-276.

69. Ribes M, Jimenez E, Yahel G, López-Sendino P, Diez B, Massana R, et al. Functional convergence of microbes associated with temperate marine sponges. Environ Microbiol. 2012;14: 1224-1239.

70. Ridley CP, Faulkner DJ, Haygood MG. Investigation of *Oscillatoria spongeliae*-dominated bacterial communities in four Dictyoceratid sponges. Appl Environ Microb. 2005;71: 7366-7375.

71. Rützler K. An unusual bluegreen alga symbiotic with two new species of Ulosa (Porifera:Hymeniacidonidae) from Carrie Bow Cay, Belize. Mar Ecol. 1981;2: 35-50.

72. Ryu T, Seridi L, Moitinho-Silva L, Oates M, Liew YJ, Mavromatis C, et al. Hologenome analysis of two marine sponges with different microbiomes. BMC Genomics. 2016;17: 158.

73. Sacristán-Soriano O, Banaigs B, Casamayor EO, Becerro MA. Exploring the links between natural products and bacterial assemblages in the sponge *Aplysina aerophoba*. Appl Environ Microb. 2011;77: 862-870.

74. Sarà M. Ultrastructural aspects of the symbiosis between two species of the genus *Aphanocapsa* (Cyanophyceae) and *Ircinia variabilis* (Demospongiae). Mar Biol. 1971;11: 214-221.

75. Schmitt S, Tsai P, Bell J, Fromont J, Ilan M, Lindquist N et al. Assessing the complex sponge microbiota: core, variable and species-specific bacterial communities in marine sponges. ISME J. 2012;6: 564-576.

76. Sipkema D, Blanch HW. Spatial distribution of bacteria associated with the marine sponge *Tethya californiana*. Mar Biol. 2010;157: 627-638.

77. Steindler L, Huchon D, Avni A, Ilan M. 16S rRNA phylogeny of sponge-associated cyanobacteria. Appl Environ Microb. 2005;71: 4127-4131.

78. Steinert G, Taylor MW, Deines P, Simister RL, De Voogd NJ, Hoggard M, et al. In four shallow and mesophotic tropical reef sponges from Guam the microbial community largely depends on host identity. PeerJ. 2016;4: e1936.

79. Sun W, Dai S, Wang G, Xie L, Jiang S, Li X. Phylogenetic diversity of bacteria associated with the marine sponge *Agelas robusta* from South China Sea. Acta Oceanol Sin. 2010;29: 65-73.

80. Tang SL, Hong MJ, Liao MH, Jane WN, Chiang PW, Chen CB, et al. Bacteria associated with an encrusting sponge (*Terpios hoshinota*) and the corals partially covered by the sponge. Environ Microbiol. 2011;13: 1179-1191.

81. Thacker RW. Impacts of Shading on Sponge-Cyanobacteria Symbioses: A Comparison between Host-Specific and Generalist Associations. Integr Comp Biol. 2005;45: 369-376.

82. Thacker RW, Starnes S. Host specificity of the symbiotic cyanobacterium *Oscillatoria spongeliae* in marine sponges, *Dysidea* spp. Mar Biol. 2003;142: 643-648.

83. Thacker RW, Diaz MC, Rützler K, Erwin PM, Kimble SJA, Pierce MJ, et al. Phylogenetic relationships among the filamentous cyanobacterial symbionts of Caribbean sponges and a comparison of photosynthetic production between sponges hosting filamentous and unicellular cyanobacteria. Custódio MR, Lôbo-Hajdu G, Hajdu E, Muricy G, editors. Porifera Research: Biodiversity, Innovation and Sustainability. Rio de Janeiro: Museu Nacional; 2007. pp 621-626.

84. Thiel V, Neulinger SC, Staufenberger T, Schmaljohann R, Imhoff JF. Spatial distribution of sponge-associated bacteria in the Mediterranean sponge *Tethya aurantium*. FEMS Microbiol Ecol. 2007;59: 47-63.

85. Thomas T, Moitinho-Silva L, Lurgi M, Björk JR, Easson C, Astudillo-García C, et al. Diversity, structure and convergent evolution of the global sponge microbiome. Nat Commun. 2016;7: 11870.

86. Topçu NE, Pérez T, Grégori G, Harmelin-Vivien M. In situ investigation of Spongia officinalis (Demospongiae) particle feeding: Coupling flow cytometry and stable isotope analysis. J Exp Mar Biol Ecol. 2010; 389:61-69.

87. Trindade-Silva AE, Rua C, Silva GG, Dutilh BE, Moreira APB, Edwards RA, et al. Taxonomic and functional microbial signatures of the endemic marine sponge *Arenosclera brasiliensis*. PLoS ONE. 2012;7: e39905.

88. Usher KM, Kuo J, Fromont J, Sutton DC. Vertical transmission of cyanobacterial symbionts in the marine sponge *Chondrilla australiensis* (Demospongiae). Hydrobiologia. 2001;461: 9-13.

89. Usher KM, Toze S, Fromont J, Kuo J, Sutton DC A new species of cyanobacterial symbiont from the marine sponge *Chondrilla nucula*. Symbiosis. 2004a;36: 183-192.

90. Usher KM, Fromont J, Sutton DC, Toze S. The biogeography and phylogeny of unicellular cyanobacterial symbionts in selected sponges from Australia and the Mediterranean. Microb Ecol. 2004b;48: 167-177.

91. Usher KM, Kuo J, Fromont J, Toze S, Sutton DC. Comparative morphology of five species of symbiotic and non-symbiotic coccoid cyanobacteria. Eur J Phycol. 2006;41: 179-188.

92. Vicente VP. Response of sponges with autotrophic endosymbionts during the coral-bleaching episode in Puerto Rico. Coral Reefs. 1990;8: 199-202.

93. Wang G, Yoon SH, Lefait E Microbial communities associated with the invasive Hawaiian sponge *Mycale armata*. ISME J. 2009;3: 374-377.

94. Wang JT, Hirose E, Hsu CM, Chen YY, Meng PJ, Chen CA. A coral-killing sponge, *Terpios hoshinota*, releases larvae harboring cyanobacterial symbionts: an implication of dispersal. Zool Stud. 2012;51: 314-320.

95. Webb VL, Maas EW. Sequence analysis of 16S rRNA gene of cyanobacteria associated with the marine sponge *Mycale* (*Carmia*) *hentscheli*. FEMS Microbiol Lett. 2002;207: 43-47.

96. Webster NS, Hill RT. The culturable microbial community of the Great Barrier Reef sponge *Rhopaloeides odorabile* is dominated by an α-Proteobacterium. Mar Biol. 2001;138: 843-851.

97. Webster NS, Luter HM, Soo, RM, Botté ES, Simister RL, Abdo D, et al. Same, same but different: symbiotic bacterial associations in GBR sponges. Front Microbiol. 2013; 3: 444.

98. Webster NS, Taylor MW, Behnam F, Lücker S, Rattei T, Whalan S., et al. Deep sequencing reveals exceptional diversity and modes of transmission for bacterial sponge symbionts. Environ Microbiol. 2010;12: 2070-2082.

99. White JR, Patel J, Ottesen A, Arce G, Blackwelder P, Lopez JV. Pyrosequencing of bacterial symbionts within *Axinella corrugata* sponges: diversity and seasonal variability. PloS ONE. 2012;7: e38204.

100. Wilkinson CR. Microbial associations in sponges. I. Ecology, physiology, and microbial populations of coral reefs sponges. Mar Biol. 1978;49: 161-167.

101. Wilkinson CR. Nutrient translocation from symbiotic cyanobacteria to coral reef sponges. In: Lévi C, Boury-Esnault N, editors. Biologie des spongiaires. Paris: Colloques Internationaux du CNRS; 1979. pp. 373-380.

102. Wilkinson CR. Cyanobacteria symbiotic in marine sponges. In: Schwemmler W, Schenck HEA, editors. Endocytobiology, Endosymbiosis and Cell Biology. Berlin: De Gruyter; 1980. pp 993-1002.

103. Wilkinson CR. Net primary productivity in coral reef sponges. Science. 1983;219: 410-412.

104. Yang Z, Li Z. Spatial distribution of prokaryotic symbionts and ammoxidation, denitrifier bacteria in marine sponge *Astrosclera willeyana*. Sci Rep UK. 2012;2: 528.

105. Yang J, Sun J, Lee OO, Wong YH, Qian P-Y. Phylogenetic diversity and community structure of sponge-associated bacteria from mangroves of the Caribbean Sea. Aquat Microb Ecol. 2011;62: 231-240.

106. Yu CH, Lu CK, Su HM, Chiang TY, Hwang CC, Liu T, et al. Draft genome of *Myxosarcina* sp. strain GI1, a baeocytous cyanobacterium associated with the marine sponge *Terpios hoshinota*. Stand Genomic Sci. 2015;10: 28.

107. Zhang F, Vicente J, Hill RT. Temporal changes in the diazotrophic bacterial communities associated with Caribbean sponges *Ircinia stroblina* and *Mycale laxissima*. Front Microbiol. 2014;5: 561.

108. Zhu P, Li Q, Wang G. Unique microbial signatures of the alien Hawaiian marine sponge *Suberites zeteki*. Microb Ecol. 2008;55: 406-414.
